# Supplementary figures and images for: Cardiac activity impacts cortical motor excitability
Source: PLoS Biol. 2023 Nov 28;21(11):e3002393. doi: 10.1371/journal.pbio.3002393 (PMC10684011; doi:10.1371/journal.pbio.3002393)

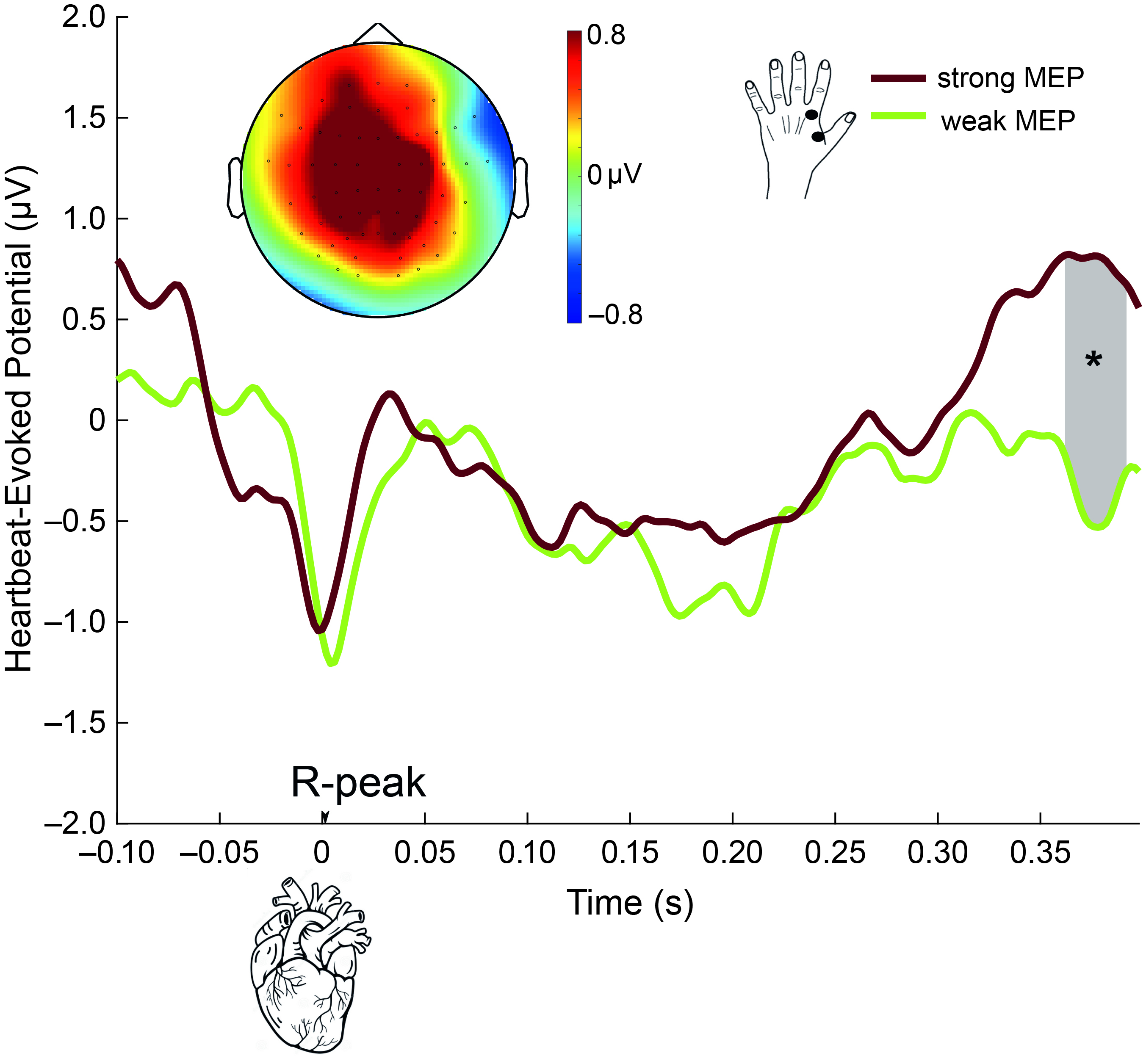

Supplement: S1 Fig — (TIF) [file pbio.3002393.s001.tif]

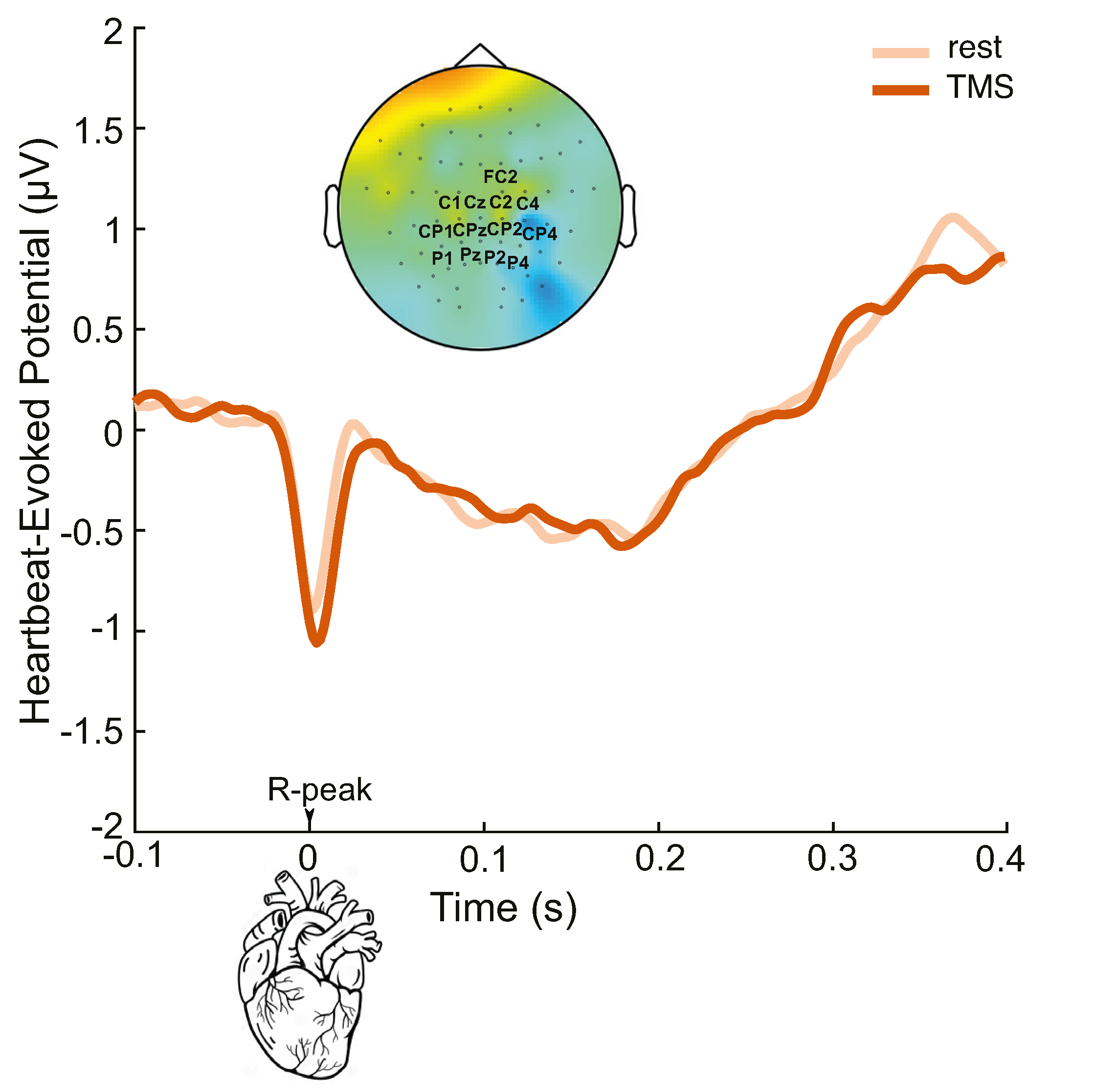

Supplement: S2 Fig — (TIF) [file pbio.3002393.s002.tif]

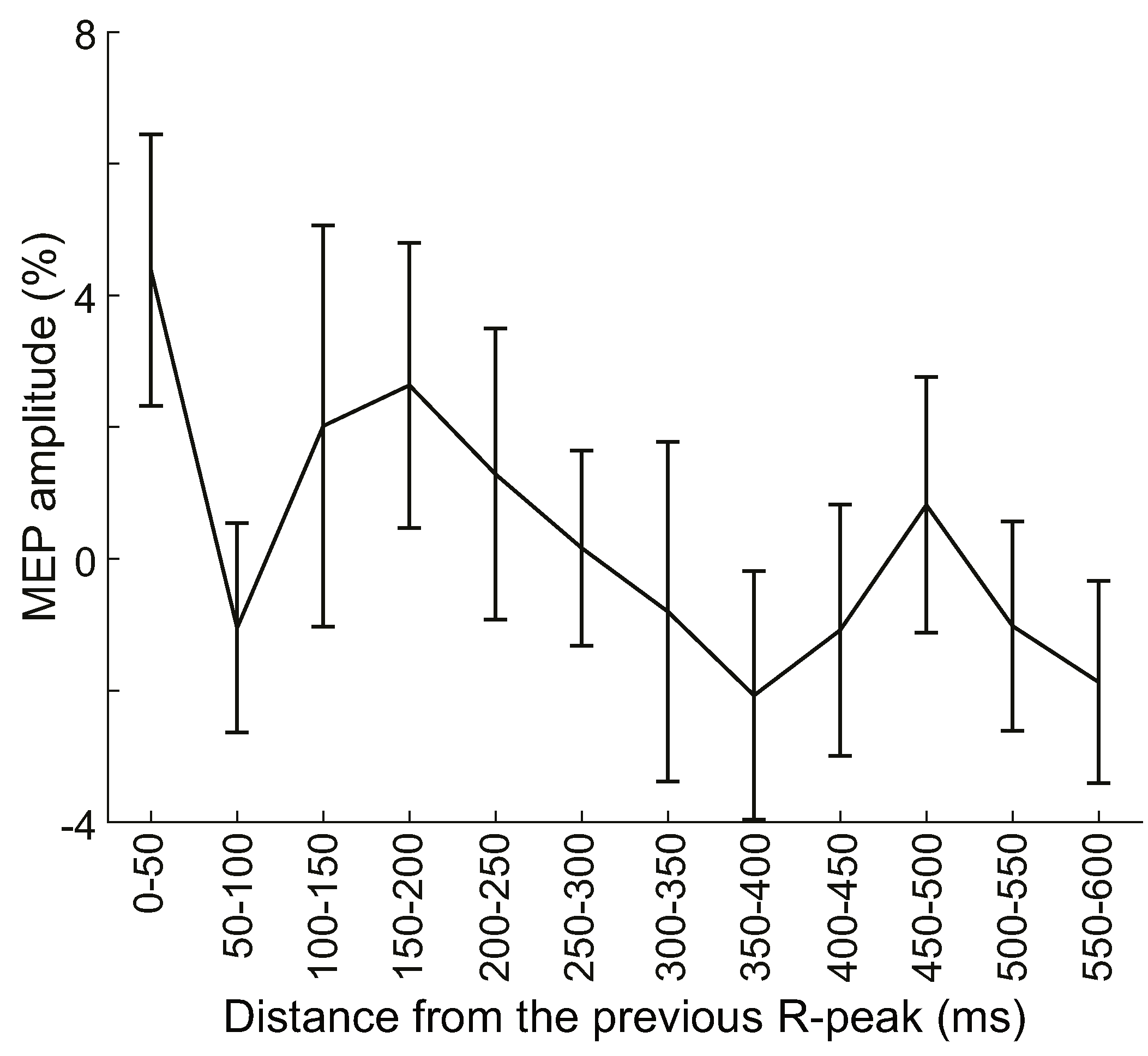

Supplement: S3 Fig — (TIF) [file pbio.3002393.s003.tif]
