# Supplementary material for: Cardiac activity impacts cortical motor excitability
Source: PLoS Biol. 2023 Nov 28;21(11):e3002393. doi: 10.1371/journal.pbio.3002393 (PMC10684011; doi:10.1371/journal.pbio.3002393)
Supplement: Attachment — Submitted filename: Review_plosbiology_round4_VN.docx [file pbio.3002393.s004.docx]

**RESPONSE LETTER TO REVIEWERS**

We thank all the reviewers and editors for taking their time and providing us their valuable feedback and constructive comments. In our efforts to enhance the clarity and improve the broad accessibility of the manuscript, we have implemented substantial adjustments. Specifically, we focused on reworking the results section to ensure that each set of analyses is preceded by a clear motivation, outlining the aim and purpose behind each analysis. Additionally, we incorporated a concise conclusion after each set of analyses to summarize the key findings (see response to **1.3**). Furthermore, we rewrote the first paragraph of the discussion to highlight the novelty of our study and to improve its accessibility (see response to **2.3**).

In what follows, the reviewers’ comments are in italics and our responses to each comment are in plain text. Quotes from the revised manuscript are printed in blue font color. In the concluding section of our responses to the reviewers, we address each of the editorial requests individually.

Reviewer #1 - Comments and Responses

1.1 *Al et al., examined whether cortical and corticospinal excitability changes as a function of the cardiac phases (systole and diastole). They examined such a potential modulation via TMS (to measure excitability), with EEG to measure TMS and heartbeat-evoked potentials, and ECG to obtain the corresponding phase-locked measures. The authors found higher excitability and greater TMS-evoked potential in the systole phase of the cardiac cycle (vs. sham condition). In addition, they also recorded EMG during a simple motor task (pinching) and examined muscle-related activity. The subsequent results indicate that EMG activity was higher circa 220-520ms after the pinch onset. If the pinch was initiated in systole, desynchronization of sensorimotor oscillations was higher in the frequency range of alpha and beta oscillations. The authors conclude that the systole phase of the cardiac cycle may be related to motor facilitation (action) whereas the diastole phase to perception.*

*I read the preprint a while ago, and overall, the authors have done an excellent job. The study involves an amalgam of techniques that is not usually found elsewhere. The manuscript addresses pertinent issues in the literature and moves forward the various fields (e.g., interoception). Overall, the authors have produced a stimulating piece of work. The following minor comments, suggestions, and clarifications may enhance the manuscript's scope and clarity. Please find below the comments by section:*

We thank the reviewer for the positive and constructive feedback. We addressed all their concerns and changed the manuscript accordingly. Please find our responses to each comment below.

*Introduction and discussion:*

1.2 *In the introduction, the authors refer to the study by Ohl et al., which demonstrated that microsaccades are coupled to the cardiac cycle. However, the way the manuscript is currently written, it seems as though two studies (references 17-18) showed this finding. In contrast, study 18 (Galvez-Pol et al.) revealed that saccades are locked around systole and visual fixations in diastole, without presenting microsaccade data. Relatedly, Galvez-Pol et al. also suggested that the diastole phase of the cardiac cycle may be linked to sensory gain (perception), which should be more accurately referenced, as the idea behind the current manuscript has been previously noted.*

*Furthermore, a recent paper (*[*https://doi.org/10.7554/eLife.78126*](https://doi.org/10.7554/eLife.78126)*) showed that human subjects freely moved their index finger to tap a key during systole, a finding that is consistent with active visual tasks without perceptual discrimination requirements (see Kunzendorf et al., 2019). The same study discovered that when participants had to sense gratings, if touch initiation occurred during systole, they extended the duration of the touch, whereas during diastole, touches were shorter. This implies that participants needed more time for perception when it began in systole or sought to reach a more sensitive phase of the cycle. This work also supports the current manuscript; however, the introduction and discussion do not sufficiently address it. By incorporating these points, the current manuscript will be better situated within the existing literature.*

Thank you for mentioning these studies. We now elaborate on them further and include new references as well.

Introduction

Furthermore, when touch was initiated during systole, the duration of active sensation was longer (Galvez-Pol, Virdee, Villacampa, & Kilner, 2022). However, for some other motor activities, a facilitatory effect of systole has been observed, e.g., self-initiated movements occurred more frequently during systole (Galvez-Pol et al., 2022; Kunzendorf et al., 2019) or around the R-peak (Palser, Glass, Fotopoulou, & Kilner, 2021); but see (Herman & Tsakiris, 2020). Similarly, an effect of the cardiac cycle was also observed in gun shooting, with shooters preferentially triggering a gun in a cardiac window that included a large part of systole and the initial part of diastole (Konttinen, Mets, Lyytinen, & Paananen, 2003); but also see an opposite effect for elite shooters (Helin, Sihvonen, & Hänninen, 1987). Furthermore, (micro)saccades occur more often during systole whereas fixations during diastole (Galvez-Pol, McConnell, & Kilner, 2020; Ohl, Wohltat, Kliegl, Pollatos, & Engbert, 2016). One potential explanation for these effects has been suggested to be related to blood movement within the body (peripheral), rather than being driven by top-down neural mechanisms (central) (Galvez-Pol et al., 2020). [page 2, line 44-54]

Discussion

This would also explain why eye movements occur more often during systole whereas eyes fixate on visual stimuli more often during diastole (Galvez-Pol et al., 2020). [page 17, line 415-417]

For example, we recently demonstrated that somatosensory percepts and their neural processing are attenuated during systole (Al et al., 2020; Al, Iliopoulos, Nikulin, & Villringer, 2021). To compensate for this reduced perceptual sensitivity, a recent study revealed that touches initiated in the systole phase were held for longer periods of time (Galvez-Pol et al., 2022). [page 17, line 400-403]

*Results:*

1.3 *My primary concern is the clarity of the rationale underpinning the analyses and the subsequent results. In its current state, the manuscript features numerous analyses with insufficient or unclear reasoning. Enhancing this aspect should not be challenging: considering the journal's broad readership, I recommend providing a more comprehensive explanation of the rationale behind the analyses that led to the results. This can be achieved by including the thought process and bits of explanations at the beginning of each subsection, particularly in the second half of the results section (e.g., IBIs and HEPs). By doing so, the manuscript's framework and the coherence of the narrative that the authors aim to present can be enhanced.*

We thank the reviewer for this critic. To enhance readability of the manuscript, we now added an explanation of the research question in the beginning of each subsection of results and at the end of the section we highlighted the main finding.

**Motor-evoked potentials change across the cardiac cycle**

We first aimed to determine whether corticospinal excitability changes systematically over the phases of the cardiac cycle. [page 6, line 119-120]

So, these results suggest that corticospinal excitability is higher during systole compared to diastole. [page 6, line 130-131]

**TMS-evoked potentials vary across the cardiac cycle**

In addition to corticospinal excitability, we also aimed to test differences of cortical responses to the TMS pulse between the systole and diastole phases of the cardiac cycle. [page 7, line 144-145]

These results show that similar to corticospinal excitability, purely cortical excitability increases during systole, suggesting increased neuronal excitability in the motor system. [page 7, line 166-167]

**Muscle-related peripheral and central activity fluctuates across the cardiac cycle**

To determine whether changes in motor excitability during systole facilitate muscle activity in the absence of TMS stimulation, we analyzed cardiac effects on muscle activity in a follow-up motor pinch task. During the task, participants were asked to pinch a dynamometer with their index finger and thumb, while we recorded EMG, EEG, and ECG signals. [page 8, line 181-184]

Thus, these results indicate that both peripheral muscle activity and its central correlates are stronger when the movement starts during systole as compared to diastole. [page 9, line 203-205]

**Heart rate changes depending on the timing of TMS across the cardiac cycle**

Previous research suggests that TMS stimulation has significant effects on heart rate (Iseger, Padberg, Kenemans, van Dijk, & Arns, 2021). However, it is not known whether this effect depends on the cardiac phase of the TMS stimulation. Therefore, in an exploratory analysis, we investigated the changes of the heart rate in response to TMS stimulation during systole and diastole. As a measure of heart rate, we calculated the length of interbeat interval in the cardiac cycles before TMS (pre-TMS), during TMS, and post-TMS stimulation. [page 10, line 219-224]

These results suggest that heart rate is influenced by TMS stimulation only when administered during systole but not during diastole. [page 11, line 242-243]

**Heartbeat-evoked potentials fluctuate depending on motor excitability levels**

The analysis of cardiac phase effects on motor excitability showed a contrasting pattern to the previous findings on somatosensory perception. Here, we asked whether a similar contrast would be observed for another aspect of heart-brain interactions, which is the cortical responses to heartbeats, so-called heartbeat-evoked potentials (HEP). Therefore, we analyzed the relationship between HEPs and motor excitability levels. [page 11, line 248-252]

Overall, these analyses show that increases in HEP amplitudes lead to stronger motor excitability levels, which again stands in contrast to the previous HEP findings in somatosensory perception. [page 12, line 276-278]

1.4 *Relatedly, it is not clear to me the rationale behind the IBIs section. Why the TMS applied to the motor area should affect distinctively the duration of heartbeats as a function of cardiac phase?*

The effect of TMS on heart rates has been a topic of previous research (Iseger et al., 2021). Here, we addressed this by looking into this relationship by including cardiac phase of the TMS. To make this point clearer, we edited the manuscript in the following way:

Previous research suggests that TMS stimulation has significant effects on heart rate (Iseger et al., 2021). However, it is not known whether this effect depends on the cardiac phase of the TMS stimulation. Therefore, in an exploratory analysis, we investigated the changes in the heart rate in response to TMS stimulation during systole and diastole across time. [page 10, line 219-222]

1.5 *Figure 1: State briefly in the text accompanying the figure that the diastole window has been equated to the length of the systole one for analyses purposes.*

We added this information in Figure 2, where we explain the definitions of systole and diastole.

To equate the probability of a stimulation event occurring in either of the two phases, equal lengths of systole and diastole were defined. [page 6, line 136-138]

1.6 *MEPs and figure 2: I suggest the authors to plot the scatter data of the MEPs with the box plots (i.e., individual dots depicting the means or medians of the subjects). If the plot changes the resolution of the axis and the difference between conditions is not easily discernible, the authors can always add a new plot of the difference by subtracting the systole and diastole MEPs (as visual aid).*

As the reviewer suggested, we added the mean of subject values in Figure 2b.

**Figure 2.** Changes in corticospinal excitability depending on the timing of TMS application across the cardiac cycle. (**a**) Schematic of the cardiac cycle. The systolic phase (indicated in red) starts with the R-peak and reflects the ventricular contraction of the heart (leading to blood ejection), whereas the diastolic phase (indicated in blue) represents the relaxation phase during which the heart refills with blood. To equate the probability of a stimulation event occurring in either of the two phases, equal lengths of systole and diastole were defined. (**b**) MEP amplitudes of the first dorsal interosseus muscle in the left hand are higher in response to TMS stimulation during systole (red) compared to diastole (blue). The individual points indicate the mean MEP amplitudes for each subject. **p*<0.05

1.7 *Figure 4: If I understand correctly, event-related desynchronization following muscle activation was computed for the frequency range between 8 and 25Hz. Were alpha and beat analysed separately or together? The analysis seems to indicate 'together' while the topoplots 'separate'. If the latter is true, I advise consistency between analysis and figures; show one topoplot instead of two.*

We agree with the reviewer. Following this suggestion, we changed the topoplot to represent 8-25 Hz frequency range.

**Figure 4.** Fluctuations of muscle-related activity depending on the pinch onset across the cardiac cycle during a motor pinch task. (**a**) Muscle force, measured by the normalized linear envelope of EMG activity, in the left hand, was significantly higher from 220 to 522 ms following pinch onset (at 0 ms) during systole compared to diastole. (**b**) Similarly, systolic and diastolic sensorimotor oscillations were analyzed in the range of 8–30 Hz in the sensorimotor electrodes to quantify event-related desynchronization following the muscle activation. Cluster statistics revealed that when subjects started the pinch during systole, the desynchronization of sensorimotor oscillations was higher in the frequency range of 8–25 Hz between 0 and 726 ms following pinch onset. The raster plot shows the contrast between systole and diastole. (**c**) The topography of the significant contrast between systole and diastole.

*Discussion:*

1.8 *Regarding the studies about shooting in systole: also note that elite rifle shooters pull the trigger during diastole, with beginners firing during either phase but with better results during diastole (Helin et al., 1987). It has been suggested that this effect is related to the mechanical movement caused by the heart's contraction (Konttinen et al., 2003).*

The reviewer is right that the results of Helin et al. (1987) suggests that shooting during diastole provides better shooting performance, however this was not replicated in Kontinnen et al., 2003. They rather found that “in air rifle shooting, the optimum firing time within the cardiac cycle may be located in the systolic phase.” Now, we refer to Helin et al. (1987) to let readers know about the different results in the literature.

Similarly, an effect of the cardiac cycle was also observed in gun shooting, with shooters preferentially triggering a gun in a cardiac window that included a large part of systole and the initial part of diastole (Konttinen et al., 2003); but also see (Helin et al., 1987). [page 2, line 48-50]

1.9 *In the discussion section, I advise the authors to write a section of Constraints on Generality (COG); see Simmons et al., 2017. This involves adding some additional information about the constraints of the current work in other scenarios, tasks, paradigms, populations, etc. (see examples in the Simmons' paper). This should not be too long. This section could be also combined with the second paragraph of the discussion (which is more centred on methodological features of the present and past studies).*

The reviewer highlights an important point. We added a paragraph about the limitations and generalizability of our study in the discussion section.

While the present study provides valuable insights into the influence of cardiac oscillations on motor excitability, there are several limitations that should be acknowledged. Firstly, the study sample consisted of young Caucasian individuals between the ages of 18-40. Although we anticipate that our findings may extend to the broader population, it is crucial for future studies to investigate the effects of cardiac oscillations in more diverse subject populations across different racial groups. Secondly, we only stimulated the right motor cortex. Further research should examine whether similar cardiac effects on motor excitability can be observed for left-hemispheric stimulation. Finally, we did not examine the potential impact of respiratory oscillations on the observed results. As respiration has been shown to organize neural dynamics (Ito et al., 2014) and modulate cortical excitability (Kluger, Balestrieri, Busch, & Gross, 2021), it would be important for future studies to investigate how respiratory phase (i.e., inhale and exhale) during TMS stimulation and movement initiation might interact with the heartbeat-related effects. [page 19, line 480-491]

1.10 *The current results can be mentioned within recent discoveries on the relationship between breathing and neural function to provide a more comprehensive understanding of the interactions between physiological processes and cognition. By incorporating (briefly) the findings on the respiratory cycle, the scope of the paper can be broadened, and the potential interactions between cardiac and respiratory cycles in modulating neural activity can be discussed. For instance, the research of Kluger et al., (2021) and Allen's review on the topic (2022) can be mentioned in this context.*

We thank the reviewer for this nice suggestion. We addressed this point in the discussion section as we indicated in our previous response (see response to 1.9)

*Methods:*

1.11 *To enhance clarity, what is the reason for the interpolation between -2 and 15ms in step 9? (Aligning the data, missing data..)*

TMS stimulation causes strong artifacts in EEG data around the stimulation event, which is known as TMS pulse artifacts. This artifact is very steep and several orders of magnitude larger than neural activity recorded with EEG and has high frequency components (Rogasch et al., 2017). To prevent it from leaking into the post stimulus time window (e.g., due to filtering), it needs to be removed from the data. In our dataset, this artifact was between -2 and 15 ms around TMS pulse. We removed the data in this time window and interpolated the EEG in this time window for visualization purposes. To clarify this point, we edited the manuscript in the following way:

We then applied a cubic interpolation (from -2 to 15ms relative to TMS stimulation) before applying the same filtering procedure (as described above) to the data (Rogasch et al., 2017). The interpolated data was only used for data visualization and excluded from statistical analyses. This way we ensured that the TMS artifacts did not smear into the post-stimulus window during the filtering process. [page 23, line 598-602]

1.12 *Independent Component Analysis (ICA) is a widely used technique for removing artifacts in EEG data. A good point is that It separates the mixed signals into statistically independent components, which can then be analyzed and classified as either neural or artifactual. I believe the implementation of ICA benefits from the data filtering (step 5) in Figure 7. How were the parameters chosen for this filter? (I thought 1Hz was recommended). Also, can the authors provide additional information about the removed ICA? (e.g., did the authors follow any rule or visual inspection? And/or how many ICs were approximately removed in average?). One issue with this sort of study is the quantity of removed data from the recording. In this context, researchers using ICA and removing TMS or cardiac artifacts may face ambiguity in component classification. In other words, identifying and classifying independent components as artifacts or neural signals can be challenging and may require expert knowledge. This can lead to false positives (removing neural signals) or false negatives (retaining artifacts). Also, ICA assumes statistical independence. ICA relies on the assumption that sources are statistically independent. If this assumption is violated, the separation of artifacts from neural signals may be less accurate.*

Following the TMS-EEG literature, we applied ICA twice (Hernandez-Pavon et al., 2023). Before the first round of ICA, no filtering was applied to prevent the TMS decay artifact to smear into the rest of the data. To eliminate the TMS decay artifact, the three largest ICs explaining the variance between -150 to 150 ms were removed (Rogasch et al., 2017). The rest of the components were forward-projected. After the decay artifact was removed in this way, copies of these datasets were kept. Then, a 4^th^ order Butterworth bandpass filter (0.5-45 Hz) was applied as we did in our earlier study (Al et al., 2020). Our previous analyses showed that a 0.5-Hz high-pass filter did not interfere with the cardiac-related neural effects. We used a 45-Hz low pass filter to reduce the high-frequency activity in the data. We additionally applied a 50-Hz notch filter (with a stopband from 45 to 55 Hz) since the EEG cabin was not completely isolated from electrical outlets due to the presence of the TMS machine. A second round of ICA was applied to determine remaining TMS, ocular, muscle and cardiac artifacts. In this analysis step, we individually checked every ICA component and if there was a doubt about a component, the analyst received a second opinion from another expert. We determined ocular and muscle IC components by their prototypical topographies. To determine ICs representing cardiac artifacts, we epoched ICA component activities in relation to R-peaks and checked for a corresponding ECG-like activity. On average, 11 ± 2 ICA components were removed in the second data cleaning round. Finally, we visualized the spectrum of each IC component to ensure that no artifacts were present in the data anymore.

We edited the methods section to give more details on the ICA-related data cleaning process and also to cite the related methodology.

The large-amplitude TMS artifacts between -2 to 8 ms were removed from each trial and then, the remaining data segments were concatenated, in line with a previously established procedure (Rogasch et al., 2017). Next, ICA (round 1) was applied using pop_runica as implemented in EEGLAB, used with the FASTICA algorithm implementing the ‘tanh’ contrast function and a symmetrical approach (Hyvärinen, 1999; Rogasch et al., 2017). To remove TMS decay artifacts, the three components explaining the most variance between -150 to 150 ms were removed and other components were forward-projected (Rogasch et al., 2017). After the decay artifact was removed in this way, copies of these datasets were kept. Then, a 4^th^ order Butterworth bandpass filter (0.5 to 45 Hz) and a 50 Hz notch filter (with a stopband from 45 to 55 Hz) were applied. A second round of ICA was applied to determine remaining TMS, ocular, muscle and cardiac artifacts. Afterwards, these ICA weights were applied to the copied dataset after the first round of ICA (unfiltered). On average 11 ± 2 artifactual components were removed and then the data was forward-projected. [page 22-23, line 585-598]

*Reviewer # 2 - Comments and Responses*

2.1 *The manuscript describe an interesting and timely study on the modulation of cortical and cortico-spinal excitability by cardiac cycle phases (systole vs. diastole). The authors test two alternative hypotheses underlying the changes in perceptual threshold and motor execution, based on earlier literature that have shown both attenuation of somatosensory perception and facilitation of motor activity during systole. Their findings support facilitatory effect on motor excitability, as they show higher central and peripheral TMS-evoked responses during systole. They also report several related evidence including effects in cardiac evoked activity and heart rate, as well as stronger muscle activity and sensorimotor cortex desynchronization during systole. The paper is generally well written, and the experiments are conducted well, with carefully planned control conditions. The number of subjects is not very big, but of needed size to allow appropriate statistical testing, with appropriate power analysis (however, see comment below related to the statistics). There are some questions, suggestions and concerns I think would need to be addressed to improve the readability and confirm the significance of the study for the field. Currently the paper is not written in a generally accessible way, and therefore it is hard to say to what extent it gives outsanding contribution. The structure and multitude of the findings are presented in rather fragmented manner and do not develop into coherent picture of a) what is the current gap (and novelty the paper brings) in the literature and b) how / why are all the different experimental manipulations and measurements needed to fill this gap.*

We thank the reviewer for the positive and constructive feedback. We addressed all their concerns and edited the manuscript accordingly. Please find our responses to each comment below.

2.2 *First, it is not entirely clear to me why are the authors forming two alternative hypotheses (e.g. Intro end of page 3), one based on earlier results on *perceptual* threshold and the other based on earlier results on *motor* execution. Their research examines only motor cortex excitability and peripheral motor evoked potentials, and thus it seems more suitable to make hypothesis based on earlier studies on the motor side. Other cardiac-related effects are of course interesting and worth reporting, but I wonder whether they should be presented as equally relevant with the motor studies. Also in the discussion the impression is given that the initial assumption would be to see similar cardiac-brain coupling for perception and action; in my understanding this is not what the earlier literature would propose - I may be mistaken but clearer and more elaborate reasoning on the background would better justify the novelty of the paper.*

Our previous experiments investigated cardiac influences on somatosensory perception, in which we observed inhibitory effects of systolic activity and heartbeat-evoked activity (Al et al., 2020, 2021). When we started this study in 2020, there were some ideas that these cardiac effects might be present due to a decrease in overall cortical excitability due to baroreceptor activity (Duschek, Werner, & Paso, 2013; Lacey & Lacey, 1970; Rau & Elbert, 2001). So, we moved from the somatosensory domain to the motor domain to investigate at excitability in a more physiological way. This is because motor evoked potentials (MEPs) are considered as one of the most direct ways to estimate cortical excitability. While one possibility was a decrease in overall excitability across the cortex (including both motor and somatosensory domains), another possibility was finding an increase in the motor domain due to previous findings suggesting increased motor activity during systole – yet this notion was not uniformly consistent across different studies. For example, in the study by Kunzendorf et al. (2019), more frequent actions occurred during systole, in another experiment actions were evenly distributed along the cardiac cycle (Herman et al., 2020). Similarly, while Helin et al. (1987) observed that beginner rifle shooters fire during either phase but with better results during diastole, Kontinnen et al., (2003) found increased firing probability during systole without a change in performance. So, there was no consensus on cardiac effects on the motor domain. Additionally, these motor related effects were suggested to occur due to blood circulation in the body, rather than reflecting a top-down neural effect (Galvez-Pol et al., 2020). If this interpretation was true, then we would not expect a change in cortical motor excitability across the cardiac cycle. After we analyzed the data and integrated a new literature, it became clearer that there might be an optimal window for action and perception. However, this was not as clear to us when we started the study.

To clarify our reasoning in our hypotheses, we edited the introduction:

An overall systolic dampening of cortical processes was suggested to be due to baroreceptor activation during systole (Duschek et al., 2013). In support of this view, reaction times to auditory, visual and tactile stimuli have been shown to be slower for systolic presentation (Edwards, Ring, McIntyre, Carroll, & Martin, 2007; Saari & Pappas, 1976). Furthermore, when touch was initiated during systole, the duration of active sensation was longer (Galvez-Pol et al., 2022). However, for some other motor activities, a facilitatory effect of systole has been observed, e.g., self-initiated movements occurred more frequently during systole (Galvez-Pol et al., 2022; Kunzendorf et al., 2019) or around the R-peak (Palser et al., 2021); but see (Herman & Tsakiris, 2020). Similarly, an effect of the cardiac cycle was also observed in gun shooting, with shooters preferentially triggering a gun in a cardiac window that included a large part of systole and the initial part of diastole (Konttinen et al., 2003); but also see an opposite effect for elite shooters (Helin et al., 1987). Furthermore, (micro)saccades occur more often during systole whereas fixations during diastole (Galvez-Pol et al., 2020; Ohl et al., 2016). One potential explanation for these effects has been suggested to be related to blood movement within the body (peripheral), rather than being driven by top-down neural mechanisms (central) (Galvez-Pol et al., 2020). [page 2, line 41-54]

2.3 *The research question is clear, but the way the different recordings, manipulations and analysis pipelines are used to answer to this question is not explained well. This gives a rather fragmented impression, for example in the list of results (different sub-titles) in the Result section, as well as beginning of discussion. Now it appears almost like the analysis has been proceeding on ad-hoc manner from one result to the next. Perhaps formulating better in the introduction the way in which (and why) each data/manipulation/analysis is planned to be used would clarify the significance of the list of results. For example, it took some time to figure out how cardiac phase-related changes in TEP's should be interpreted together with heart-evoked responses, where the phase information is lost (and all the responses are similarly phase-locked to heartbeat) - or with effects in heart rate. In general, the paper appears like not fully 'matured' to a form where its scientific significance would be easily grasped. This is especially problematic from the perspective of 'non-expert' readers (or readers from other fields).*

We thank the reviewer for this critic. As we found cardiac phase and HEP related effects on somatosensory perception, here, we aimed to test corresponding questions for motor excitability and motor activity. These analyses were hypothesis-driven. However, the analysis of heart rate changes following TMS stimulation was exploratory. To improve the readability of the manuscript, we now added an explanation of the research question in the beginning of each subsection of results and at the end of the section we highlighted the main finding. Additionally, we revised the first paragraph of the discussion.

**Motor-evoked potentials change across the cardiac cycle**

We first aimed to determine whether corticospinal excitability changes systematically over the phases of the cardiac cycle. [page 6, line 119-120]

So, these results suggest that corticospinal excitability is higher during systole compared to diastole. [page 6, line 130-131]

**TMS-evoked potentials vary across the cardiac cycle**

In addition to corticospinal excitability, we also aimed to test differences of cortical responses to the TMS pulse between the systole and diastole phases of the cardiac cycle. [page 7, line 144-145]

These results show that similar to corticospinal excitability, purely cortical excitability increases during systole, suggesting increased neuronal excitability in the motor system. [page 7, line 166-167]

**Muscle-related peripheral and central activity fluctuates across the cardiac cycle**

To determine whether changes in motor excitability during systole facilitate muscle activity in the absence of TMS stimulation, we analyzed cardiac effects on muscle activity in a follow-up motor pinch task. During the task, participants were asked to pinch a dynamometer with their index finger and thumb, while we recorded EMG, EEG, and ECG signals. [page 8, line 181-184]

Thus, these results indicate that both peripheral muscle activity and its central correlates are stronger when the movement starts during systole as compared to diastole. [page 9, line 203-205]

**Heart rate changes depending on the timing of TMS across the cardiac cycle**

Previous research suggests that TMS stimulation has significant effects on heart rate (Iseger et al., 2021). However, it is not known whether this effect depends on the cardiac phase of the TMS stimulation. Therefore, in an exploratory analysis, we investigated the changes of the heart rate in response to TMS stimulation during systole and diastole. As a measure of heart rate, we calculated the length of interbeat interval in the cardiac cycles before TMS (pre-TMS), during TMS, and post-TMS stimulation. [page 10, line 219-224]

These results suggest that heart rate is influenced by TMS stimulation only when administered during systole but not during diastole. [page 11, line 242-243]

**Heartbeat-evoked potentials fluctuate depending on motor excitability levels**

The analysis of cardiac phase effects on motor excitability showed a contrasting pattern to the previous findings on somatosensory perception. Here, we asked whether a similar contrast would be observed for another aspect of heart-brain interactions, which is the cortical responses to heartbeats, so-called heartbeat-evoked potentials (HEP). Therefore, we analyzed the relationship between HEPs and motor excitability levels. [page 11, line 248-252]

Overall, these analyses show that increases in HEP amplitudes lead to stronger motor excitability levels, which again stands in contrast to the previous HEP findings in somatosensory perception. [page 12, line 276-278]

Discussion

Using simultaneous recordings of brain activity, heart activity, and muscle activity, this study discovered that the timing of heartbeats and their neural processing were linked to changes in the excitability of the motor system. Our findings build upon previous evidence showing that somatosensory perception is affected during the systolic phase of the cardiac cycle and when heartbeats evoke stronger cortical responses. To delve deeper into these cardiac effects, we examined whether they arise from overall changes in cortical excitability. For this we performed simultaneous recordings of cortical and corticospinal excitability using electroencephalography (EEG) and electromyography (EMG) responses to transcranial magnetic stimulation (TMS), along with concurrent monitoring of cardiac activity using electrocardiography (ECG). As a result we observed multiple complementary effects of cardiac signals on motor activity. Specifically, we discovered that cortical and corticospinal excitability reached their highest levels during systole and were enhanced following stronger neural responses to heartbeats. Additionally, in a motor task, we observed that hand-muscle activity and the associated desynchronization of sensorimotor oscillations were more pronounced during systole. These findings suggest that systolic cardiac signals exert a facilitatory effect on motor excitability, which contrasts with the sensory attenuation previously reported in somatosensory perception. Therefore, our study sheds light on the existence of distinct time windows across the cardiac cycle that potentially optimize perception and action. [page 14, line 315-333]

2.4 *The introduction is very concise, and although it reviews the relevant literature related to cardiac-brain coupling, especially the means to measure cortical excitability is introduced in rather superficial and one-sided manner. There are some recent critiques (or at least needs for caution) presented for the straightforward interpretation of TEP's as an index of cortical excitability (especially for the early time-windows, see eg. Hernandez-Pavon et al., 2023). It would provide a more solid basis for the used methodology if the characteristics and possible reservations in using TEPs as indicators of cortical excitability would be good to at least mention in the introduction. This way also the following description of the control conditions in the result section would make more sense for the reader.*

We thank the reviewer for providing this valuable suggestion. In order to incorporate this suggestion, we have made the following edits to the manuscript:

Introduction

Lastly, these previous studies only included peripheral measures of corticospinal excitability without direct measurement of cortical excitability via concurrent electroencephalographic (EEG) recordings. This approach requires considering multiple concomitant factors to minimize TMS artifacts (see a recent review (Hernandez-Pavon et al., 2023). To address this limitation, stimulation intensity needs to be minimized by carefully determining the motor threshold, the effects of TMS clicks should be minimized to avoid acoustic stimulation, the experiment should have a sham condition to account for bone-conducted sound from the coil vibration (Nikouline, Ruohonen, & Ilmoniemi, 1999) and thorough data cleaning to remove TMS artifacts using spatial (e.g., ICA) and temporal filters. [page 3, line 72-80]

Results

By focusing on this time window, we prevented a possible contamination of cranial muscle artifacts in the very early peaks as well as sensory-evoked potentials in the later time windows (Hernandez-Pavon et al., 2023). [page 7, line 148-150]

2.5 *Related to the above, Result section does not give enough info about the SHAM condition (how/why was it done) for integrating it to the general picture.*

We thank the reviewer for this suggestion. As they suggested, we edited the manuscript.

To test whether these results were indeed related to neural activity of the cortex, rather than reflecting TMS- and cardiac-artifacts, we contrasted them with the sham TMS condition. During the sham condition, a plastic block between the coil and the participant’s head was placed to keep air- and bone-conducted auditory and somatosensory sensations similar to the real TMS (Nikouline et al., 1999). Additionally, pulse-related artifacts in the EEG were expected to be similar to the real TMS stimulation. Therefore, if the cardiac cycle effect on TEPs is a genuine modulation of evoked neuronal responses e by TMS, it should not be present in the sham condition. [page 7, line 152-159]

2.6 *Figure 1 gives helpful info about the methods for the reader to understand the results, but it could be better organized to help grasping the different parts of the experiment (cf. 3rd paragraph above). Moreover, it could be more informative if the timelines of the EEG, ECG and EMG would be synchronized (to understand their temporal relationships) and timeline should be added. A combined or synchronized timeline would also help to estimate the overlap of artefacts/responses.*

As suggested by the reviewer, we edited Figure 1.

**Figure 1.** Experimental Paradigm. (**a**) TMS was applied over the right primary motor cortex of the subjects. The motor response to TMS in their left hand, i.e., motor-evoked potential (MEP), was measured by bipolar electromyography (EMG). Their cortical responses to TMS, the TMS-evoked potential (TEP), as well as to heartbeats, the heartbeat-evoked potential (HEP), were measured using multichannel electroencephalography (EEG). The heart activity was recorded via electrocardiography (ECG). (**b**) After determining the individual resting motor threshold (RMT), subjects underwent a resting-state EEG measurement. Thereafter, 416 single TMS pulses with an intensity of 120% of the RMT were applied in four blocks. There were also four blocks of sham conditions, in which a plastic block was placed between the TMS coil and the head of the subject. The pairs of real and sham TMS blocks were randomized across the subjects. At the end of the TMS blocks, participants performed a motor pinch task. In this task, they were instructed to squeeze a pinch gauge with their left thumb against the index finger while a red circle was presented in the middle of the monitor. When the circle became green, they relaxed their fingers. In this order, subjects performed thirty trials.

2.7 *What is the reason for presenting the correlational analysis of MEP and TEP amplitude differences across cardiac cycle first as 'initial analysis' and the following analysis of the cardiac changes in MEP against the modulation of earlier TEP's as 'exploratory'. When reading the results, it is not clear what is the difference between these analysis approaches and/or what is the particular research questions they respond to.*

This analysis was done to investigate whether MEP and TEP modulation across the cardiac cycle correlated with each other. In other words, we tested whether the same mechanism underlies the changes in corticospinal and cortical excitability between systole and diastole. However, the reviewer is right. This analysis might be confusing for the reader and it is not so informative. For simplicity reasons, we removed this exploratory analysis from the manuscript.

2.8 *As a detail (and a topic outside of my expertise): the blood-circulation -related contribution to the cardiac cycle effect on pinching-related EMG envelope is controlled by the analysis of rest condition (?) - is there a possibility that this does not properly represent the situation during muscle activity, and would this influence to the interpretation? In other words, can it be that the blood-circulation related modulation is different (more prominent) when muscles are activated, and thus the contrasting of diastole vs. systole during rest is not a valid control condition to rule out this impact?*

Thank you for raising this concern. Since there is no movement and thus no EMG in the resting state, the additional analysis during the resting state was performed only in order to control for the possibility of a residual ECG artifact to produce differences in the EMG. Certainly blood perfusion in muscles is different depending on whether the muscle is at rest of active (Nguyen & Davidson, 2019). It is important to mention here that we do not equate resting state and task conditions as they are clearly different in terms of hemodynamics. On the basis of the resting-state analysis where we showed that cortical excitability is increased during systole, we rather conjectured that such excitability changes should be translated to the corresponding changes in the motor output. And this is indeed what we observed. Since we did not apply TMS during the task, the question remained to what extent cardiac-cycle changes in excitability are different during rest and task. This warrants future studies on this topic.

2.9 *Results related to the heart rate changes; some more details should be given for interpreting the results; from what time-window was the heart rate calculated (and what measure was used? Heart acceleration or interval between beats? Where is the analysis described for the heart rate related measures? Authors controlled for the possible artefacts (auditory, somatosensory) on the effects on heart rate by adjusting the heart rate with the one during sham TMS condition; why was this procedure chosen and not conducting the similar analysis for the TMS condition (i.e. time x diastole/systole)? Or is this what authors mean by adjusting?*

We thank the reviewer for pointing out a possible confusion regarding our heart rate analysis. The effect of TMS on heart rates has been a topic of research (Iseger et al., 2021). Here, we addressed this by looking into this relationship by including cardiac phase relating to TMS pulse. To be able to understand whether heart rate accelerates or decelerates due to TMS stimulation during systole and diastole, we calculated the length of interbeat intervals during which TMS occurred for each cardiac phase. We compared this interbeat interval to the length of the previous and following interbeat intervals (pre-TMS and post-TMS). Also, the reviewer is right that we applied the same approach to control for possible cardiac artifacts while using sham correction in our analyses.

To make this point clearer, we edited the manuscript:

Results

Previous research suggests that TMS stimulation has significant effects on heart rate (Iseger et al., 2021). However, it is not known whether this effect depends on the cardiac phase of the TMS stimulation. Therefore, in an exploratory analysis, we investigated the changes in the heart rate in response to TMS stimulation during systole and diastole across time. As a measure of heart rate, we calculated the length of interbeat intervals in the cardiac cycles before TMS (pre-TMS), during TMS, and post-TMS stimulation. [page 10, line 219-224]

Methods

Pre- and post-TMS changes in heart rate for stimulation during systole and diastole were evaluated using within-subject ANOVAs (ezANOVA function in R, v 1.3.1093, (Lawrence, 2016; R Development Core Team, 2021), in which heart rate was the dependent variable and time (pre-TMS, TMS, post-TMS) as well as cardiac phase (systole, diastole) were independent variables. [page 27, line 712-716]

2.10 *Results regarding the HEP & MEP analysis; what was the way of categorizing trials to strong vs. weak MEP (the cut point would be useful to mention in the results).*

We used MEPs for sorting since MEPs are observable at single trial level whereas this is not the case for HEP amplitudes. We sorted trials into 3 bins since this allowed us to have a strong contrast between weak and strong MEPs while providing a sufficiently high number of trials in each category. We made the following revisions to the manuscript to provide further clarification on this point:

Results

For this purpose, we sorted individual trials based on their MEP amplitudes, ranging from weak to strong, and divided them into three equal bins for each participant. The sorting was based on MEP amplitudes since they were present in every single trial included in the analysis. Subsequently, we compared the prestimulus HEP amplitudes preceding weak (1^st^ bin) and strong (3^rd^ bin) MEP levels. [page 12, line 256-260]

Methods

For statistical testing of HEP activity relating to motor excitability, we first sorted single trials according to their MEP amplitudes and split them into three equal bins for each participant. We specifically used three bins to create a strong contrast between weak and strong MEPs while providing a sufficiently high number of trials in each category. [page 27, line 716-719]

2.11 *The results (particularly for heart rate changes depending on the timing of TMS and HEP fluctuation depending on motor excitability levels) describe a rather complicated scenario with several dependencies between the TMS pulse, cortical responses and motor evoked potential - it would be really helpful for the reader to get some illustration to support the following of the analysis/results. Along the same lines, it would also guide the reading (and help to confirm the logic of the analysis) if the introduction would give at least a rough outline of the planned examination (this is mentioned also earlier). Now the result section progresses in somewhat unpredictable (but apparently meaningful) steps; for example categorizing the separate sections based on the specific part of the research question they respond to would help to keep track of the results (together with the illustration).*

We greatly appreciate the reviewer's valuable comment, which has assisted us in organizing our manuscript to enhance its accessibility. We have made updates to the manuscript by incorporating an explanation of the research question/aim at the beginning of each subsection within the Results section. Additionally, we have included a summary of the main finding at the end of the subsection to emphasize its significance. (please see our answer to *2.3*).

Additionally, we edited the introduction section to prepare the reader for upcoming analyses related to the heart rate related part, which was missing in the initial version of the manuscript:

Introduction

In addition, in an exploratory analysis, we found a specific influence of TMS on heart rate depending on the cardiac phase, which could be relevant for clinical studies. [page 4, line 96-98]

2.12 *Last result section, regarding the control analysis, do I understand correctly that this analysis is based on the known motion related to cardiac phases or was the analysis done based on the current data?*

Since points 2.12 and 2.13 relate to each other, we address them together here. Previous research has demonstrated an inverse U-shaped pattern of brain movement throughout the cardiac cycle, with motor areas reaching their closest proximity to the skull at approximately 450-500 ms after the R-peak (Zhong et al., 2009). The induced electric field of a TMS pulse is stronger with a shorter distance between the coil and brain (Hannula & Ilmoniemi, 2017). If the observed heart phase-dependent modulation of MEPs were merely attributed to a movement of the cortical tissue towards the skull, a similar temporal effect pattern would be observed in our findings. However, in our study, MEP amplitudes decreased rather linearly across the cardiac cycle, and thus differently compared to what would be expected due to cortical movement. This result indicates that the changes in MEP amplitudes are not likely to be explained by the displacement of the cortex across the cardiac cycle. We edited the manuscript to explain this reasoning:

Next, we investigated whether our findings of larger motor excitability during systole might have been related to the displacement of the cortex due to blood hemodynamics (as a result of cardiac activity). Previous research has shown that the mechanical displacement of the cortex follows an inverse u-shaped pattern across the cardiac cycle, and reaching its maximum at 450-500 ms after the R-peak (Zhong et al., 2009). As the electric field induced by a TMS pulse is stronger when the coil is closer to the brain (Hannula & Ilmoniemi, 2017), any heart phase-dependent modulation of MEPs could potentially be influenced by cortical tissue movement towards the skull. . If this was the case, we would expect a stronger TMS stimulation at the peak of the displacement. Therefore, we examined whether our findings displayed a similar temporal pattern as observed in the displacement of cortical tissue to shed light on this aspect. [page 13, line 291-300]

2.13 *I am not fully following the reasoning why MEP would be expected to follow a similar pattern as the movement of the brain, if the TMS-effects were due to the movement?*

Please see our response to 2.12.

2.14 *The beginning of discussion gives a nice overview on the results, but here again it may not be self-evident for the reader why and how the motor pinch task-related cardiac-related changes in muscle activity and cortical descynronization and especially the TMS-triggered heart-rate changes are linked to the original excitability changes. In my view, too much expert knowledge is expected form the reader to understand the value and meaning of the findings for the field. What is the phenomenon on which this paper brings the outstanding contribution? Why is it interesting and to what scientific debate or discussion does it contribute to? Occasionally the paper stays at the level of 'technically neat and comprehensive list of results'.*

We appreciate the reviewer's feedback regarding the accessibility of our discussion section. Upon reconsideration, we recognized that the initial paragraph contained technical language that might hinder comprehension for a general audience. Therefore, we have revised the paragraph to enhance its accessibility:

Using simultaneous recordings of brain activity, heart activity, and muscle activity, this study discovered that the timing of heartbeats and their neural processing were linked to changes in the excitability of the motor system. Our findings build upon previous evidence showing that somatosensory perception is affected during the systolic phase of the cardiac cycle and when heartbeats evoke stronger cortical responses. To delve deeper into these cardiac effects, we examined whether they arise from overall changes in cortical excitability. For this we performed simultaneous recordings of cortical and corticospinal excitability using electroencephalography (EEG) and electromyography (EMG) responses to transcranial magnetic stimulation (TMS), along with concurrent monitoring of cardiac activity using electrocardiography (ECG). As a result we observed multiple complementary effects of cardiac signals on motor activity. Specifically, we discovered that cortical and corticospinal excitability reached their highest levels during systole and were enhanced following stronger neural responses to heartbeats. Additionally, in a motor task, we observed that hand-muscle activity and the associated desynchronization of sensorimotor oscillations were more pronounced during systole. These findings suggest that systolic cardiac signals exert a facilitatory effect on motor excitability, which contrasts with the sensory attenuation previously reported in somatosensory perception. Therefore, our study sheds light on the existence of distinct time windows across the cardiac cycle that potentially optimize perception and action. [page 14, line 315-333]

2.15 *Although not specifically my own field, I wonder how the cardiac cycle influences to the ability of (peripheral) muscles to contract, and how would this influence to the interpretation of the current results?*

This is a very good question. It is possible that cardiac cycle might have peripheral effects on muscle contraction. We tried to control this by looking into peripheral effects (such as pulse artifacts on EMG electrode) during either sham or rest condition and subtracted them. However, we acknowledge that active muscle movement might be different than the rest condition. However, in addition to peripheral measures, we also measured cortical excitability using TEPs, which should not be affected by such peripheral effects on muscle activity. The analysis of TEP across the cardiac cycle confirmed the results of MEPs. Furthermore, during the pinch task, in addition to the muscle activity, we analyzed sensorimotor oscillations, which again reflect central neuronal changes across the cardiac cycle. Taking the evidence from all these observation levels together, we therefore conclude that the cardiac cycle effects on peripheral motor behavior originated from central nervous processes.

2.16 *In Discussion p. 16 the authors present nice reasoning on how the cardiac modulation of perception vs. action could be linked to each other. Do I understand correctly that the authors suggest that the driving force for both the effects in perception and motor activity arise from the need to dampen the rhythmic cardiac signals not to enter conscious perception? This explanation, building on the idea of 'noise cancellation', would naturally explain why there would be any need for rhythmic variation in the perceptual thresholds and motor activity with such a short time-scale. What if this assumption is not correct? Are there alternative ways to explain the effects and how does this reasoning link with the current literature and other speculations on the heart-brain coupling? For better linking with the current literature, it would be important to present the current findings & suggested hypotheses in the broader literature on the topic - the body-brain coupling is a very active field at the moment and there are many recent papers and reviews on the topic.*

The reviewer accurately highlights that we interpret our findings within the framework of interoceptive predictive coding, which explains the differences observed in perception and motor activity. Notably, recent work by Galvez-Pol et al. (2022) demonstrates that individuals actively allocate more attention to sensory perception during systole, the cardiac phase associated with lower perceptual sensitivity. These results align with interoceptive inference accounts, suggesting that we dynamically adjust the acquisition of sensory information based on our internal bodily cycles.

An alternative hypothesis of the earlier studies was the baroreceptor hypothesis, proposing that afferent neural signals from the baroreceptors occurring at systole attenuate cortical excitability and impair concurrent brain processing (Lacey & Lacey, 1970). Some studies supported this idea by showing that neck-cuff baroreceptor stimulation leads to globally reduced cortical excitability (Duschek et al., 2013; Rau & Elbert, 2001), and, consequently, to decreased muscle tone (Dworkin et al., 1994), dampened pain sensitivity (Droste et al., 1994), and reduced startle reflex (Nyklíček, Wijnen, & Rau, 2005). This evidence suggested a general inhibitory effect on neuronal excitability caused by baroreceptor afferents in a systematic manner (Duschek et al., 2013). Here, we evaluated this suggestion as an alternative hypothesis and tested whether systolic signals are associated with overall cortical excitability. Contrary to the prediction of the baroreceptor theory, we did not observe a global attenuation of cortical excitability during systole; instead, we found increased motor excitability during this phase. These findings highlight the complexity of the heart-brain coupling and raise questions about alternative mechanisms underlying the observed effects. The field of body-brain coupling is rapidly evolving, and we acknowledge the importance of placing our findings in a broader context. To better situate our results within the current literature, we incorporated relevant studies and hypotheses on body-brain coupling in the revised manuscript.

Introduction

An overall systolic dampening of cortical processes was suggested to be due to baroreceptor activation during systole (Duschek et al., 2013). In support of this view, reaction times to auditory, visual and tactile stimuli have been shown to be slower for systolic presentation (Edwards et al., 2007; Saari & Pappas, 1976). Furthermore, when touch was initiated during systole, the duration of active sensation was longer (Galvez-Pol et al., 2022). However, for some other motor activities, a facilitatory effect of systole has been observed, e.g., self-initiated movements occurred more frequently during systole (Galvez-Pol et al., 2022; Kunzendorf et al., 2019) or around the R-peak (Palser et al., 2021); but see (Herman & Tsakiris, 2020). Similarly, an effect of the cardiac cycle was also observed in gun shooting, with shooters preferentially triggering a gun in a cardiac window that included a large part of systole and the initial part of diastole (Konttinen et al., 2003); but also see an opposite effect for elite shooters (Helin et al., 1987). Furthermore, (micro)saccades occur more often during systole whereas fixations during diastole (Galvez-Pol et al., 2020; Ohl et al., 2016). One potential explanation for these effects has been suggested to be related to blood movement within the body (peripheral), rather than being driven by top-down neural mechanisms (central) (Galvez-Pol et al., 2020). [page 2-3, line 41-54]

Discussion

It is also important to note that different cardiac effects occur within the systolic and diastolic cardiac phases. During systole, in response to changes in blood pressure, baroreceptors become maximally active approximately 300 ms after R-peak (Rae et al., 2020). This change in baroreceptor activity has been suggested to be the driving force of the cardiac effects on perception. For example, during this time window, the processing of somatosensory and pain stimuli is shown to be minimal (Al et al., 2020, 2021; Edwards, Inui, Ring, Wang, & Kakigi, 2008). These findings were in line with the idea that activation of baroreceptors cause an overall decrease in cortical excitability (Duschek et al., 2013; Lacey & Lacey, 1970; Rau & Elbert, 2001). However, in the current study, we observed that motor excitability was strongest during the pre-ejection period of the systole, before arterial pressure starts to increase and activate baroreceptors. Therefore, the cardiac-related effects on the motor domain might not be mediated by baroreceptor activity, but via a direct neural pathway involving cardiac afferent neurons that fire around the R-wave and have a fast conduction velocity leading to a rapid cortical activation (Park & Blanke, 2019). Future studies could investigate this idea by using animal models. [page 17-18, line 421-437]

2.17 *Authors also speculate their findings could reflect a direct neural pathway via cardiac afferent neurons. This is an interesting suggestion for further studies, but it contrasts with the very narrow background on anatomical (and animal model) research presented in the introduction. What is currently known about the cardiac-brain connections? Are there studies supporting direct connections?*

Cardiac neurons are an integral part of the intrinsic cardiac nervous system (ICNS), a network of neurons located within the heart itself. Their primary function is to regulate the heart's electrical activity and contractility (Park & Blanke, 2019). The activity of ICNS neurons is integrated with two other levels of heart-regulating centers namely with CNS neurons (spinal cord and medulla) and with extracardial-intrathoracic neuronal pool (Dusi & Ardell, 2020). ICNS neurons receive inputs from mechano- and chemoreceptors within the heart and transmit signals to coordinate heart rate, and other cardiac functions. The firing of cardiac neurons occurs at specific phases of the ECG signal, such as around the R or T-waves, depending on their location and transduction characteristics (Park & Blanke, 2019). The myelinated and unmyelinated afferents from cardiac neurons are relayed to the nucleus of the solitary tract, through either the vagal nerve or the spinal cord (Shepherd, 1985). From there, cardiac afferents project to viscerosensory cortices, including the insula, amygdala, and cingulate cortex, through the thalamus (Park & Blanke, 2019). Park et al. proposed that these neurons contribute to direct and fast cardiac signaling to brain (Park & Blanke, 2019) since cardiac event-related potentials in rats (corresponding to HEPs in humans) could be observed 25-65 ms after the R peak (Li et al., 2015). However, given a tight integration of ICNS neurons with other abovementioned heart-regulating centers, further research is necessary to fully investigate their role in shaping heart-brain interactions. To elaborate on this point, we edited the manuscript in the following way:

Therefore, the cardiac-related effects on the motor domain might not be mediated primarily by baroreceptor activity, but rather via a direct neural pathway involving cardiac afferent neurons. These neurons receive inputs from sensory receptors within the heart and transmit signals to coordinate heart rate and other cardiac functions. They fire at specific phases of the ECG signal, such as around the R or T-waves, depending on their location and transduction characteristics. Importantly, they have a fast conduction velocity leading to a rapid cortical activation (Park & Blanke, 2019). Future studies could investigate this idea by using animal models. [page 18, line 430-437]

2.18 *Methods section gives detailed protocols for each data / analysis type, but it remains unclear which part of these analysis steps are according to golden standard principles/established (and published) protocols. Whenever possible, it would help if the authors cited any available papers describing the pipelines or principles that are followed in the analysis.*

In addition to other cited papers, we now added two important papers for analysis of MEPs(Rossini et al., 1994) and TEPs (Rogasch et al., 2017) as references.

Then, the resting motor threshold was determined as the lowest TMS intensity at which 5 out of 10 trials yielded a motor response greater than 50 µV (peak-to-peak amplitude)(Rossini et al., 1994). [page 21, line 527-529]

A successful TMS stimulation is expected to trigger a motor-evoked potential higher than 50 μV whereas a successful sham application to trigger no motor evoked activity(Rossini et al., 1994), while having other features of stimulations such as auditory click and bone vibration. [page22, line 576-579]

EEG data was first segmented between -1400 and 1000 ms around TMS stimulations. Then, the baseline correction was performed using -110 to -10ms prestimulus window. The large-amplitude TMS artifacts between -2 to 8 ms were removed from each trial and then the remaining data segments were concatenated, in line with a previously established procedure (Rogasch et al., 2017). Then, ICA (round 1) was applied using pop_runica as implemented in EEGLAB, used with the FASTICA algorithm implementing ‘tanh’ contrast function and a symmetrical approach (Hyvärinen, 1999; Rogasch et al., 2017). To remove TMS decay artifacts, the three components explaining the most variance between -150 to 150 ms were removed and other components were forward-projected (Rogasch et al., 2017). After the decay artifact was removed in this way, copies of these datasets were kept. Then, a 4^th^ order Butterworth bandpass filter (0.5 to 45 Hz) and a 50 Hz notch filter (with a stopband from 45 to 55 Hz) were applied. A second round of ICA was applied to determine remaining TMS, ocular, muscle and cardiac artifacts. Afterwards, these ICA weights were applied to the copied dataset after the first round of ICA (unfiltered). On average 11 ± 2 artifactual components were removed and then the data was forward-projected. [page 22-23, line 584-597]

2.19 *As a detail, in p. 22, line 565 onwards, authors say that only trials in which TMS stimulation triggered high-enough motor-evoked potential were included, and for sham only trials in which no evoked motor activity was observed were included. Why was this the case? It would help (especially the non-expert reader) to remind where were these categories/contrasts used in the analysis.*

A successful TMS stimulation is expected to elicit a motor-evoked potential (MEP) with an amplitude higher than 50 μV, indicating a measurable motor response. Conversely, a successful sham application should not elicit any motor-evoked activity and in general not to stimulate brain while having other features of stimulations such as auditory click, bone vibration. In order to meet such criteria, we included only corresponding TMS trials and sham trials in our analysis. Specifically, these categories were used to differentiate between trials with significant MEPs and trials with no evoked motor activity. This allowed us to examine the effects of TMS stimulation and sham application on motor responses. We revised the manuscript and moved this inclusion explanation under the *Motor-Evoked Potentials* subsection of the *Methods* part. Please see it below:

A successful TMS stimulation is expected to trigger a motor-evoked potential higher than 50 μV whereas a successful sham application to trigger no motor evoked activity(Rossini et al., 1994), while having other features of stimulations such as auditory click and bone vibration. We applied these criteria to ensure only valid TMS and sham trials were included in the analysis. This yielded on average 412 TMS trials and 405 sham trials per subject. [page 22, line 576-581]

2.20 *In p. 23, Source Analysis, line 614, authors say that individual source data were projected to the template brain, and their absolute values were used in group averages - what group averages are referred to?*

For source analyses, after projecting individual source data to the ICBM152 template, we calculated absolute values for each contrast of individual subjects. Then, we calculated a grand average for each contrast using these subject values.

Individual source data were then projected to the ICBM152 template (Fonov, Evans, McKinstry, Almli, & Collins, 2009). Following this for each subject the absolute values for each contrast were calculated. These absolute values were then used to compute grand averages of the HEP and TEP. [page 25 , line 653-656]

2.21 *Several different statistical tests are used for various time-windows, contrasts etc. How is the problem of multiple testing  (also across different data types/contrasts) dealt with? The power analysis is only reported for limited tests, but given the numerous different data and manipulations, it may not be sufficient to guarantee rigorous statistical approach. In its current form, it is also difficult to see to what extent is the analysis based on clear hypothesis and to what extent is it more of exploratory nature.*

We thank the reviewer for raising this important point regarding the statistical analysis in our study. We have revised the manuscript to provide clearer explanations regarding the distinction between hypothesis-driven analyses and exploratory analyses. We now explicitly state the hypotheses tested in each analysis, clearly outlining our primary research questions and if an analysis was exploratory, we now clearly indicate it (see the response to *2.3*).

To account for the multiple comparisons in the hypothesis-driven EEG analyses, we used cluster statistics. Additionally, this feedback helped us to notice some missing reports of effect sizes in the ANOVAs, which we now added:

The analysis showed a main effect of time (*F*2, 70=23.11, *p*=2⋅10^-8^, *ges*=2⋅10^-4^) and an interaction of time and cardiac phase (*F*2, 70=10.30, *p*=1⋅10^-4^, *ges*=2⋅10^-4^) on heart rate. [page 10, line 224-226]

This analysis again showed a similar main effect of time (*F*2, 70=7.42, *p*=1⋅10^-3^, *ges*=4⋅10^-3^) and an interaction of time and cardiac phase (*F*1.56, 54.64=3.88, *p*=0.03, *ges*=3⋅10^-3^) [page 11, line 240-242]

*Reviewer # 3 - Comments and Responses*

3.1 *In the current study, the authors investigated the influence of cardiac cycles (systole and diastole) on corticospinal and cortical excitability and on muscle activity during contraction. They also tested the effect of transcranial magnetic stimulation on cardiac measures and the relationship between heartbeat-effect cortical activity and corticospinal excitability. Contrary to the literature, the authors found greater corticospinal and cortical activity, as well as greater muscle activity during a specific time-window of pinch movement, during systole in comparison to diastole. Overall, this study brings further information regarding the relationship between cardiac activity and cortical and corticospinal excitability. However, it does not provide evidence of functional effects.

    Please find general remarks and minor comments below.*

*We thank Florent Lebon for the constructive comments and suggestions.*

3.2 *In the abstract, the authors wrote that "Cortical and corticospinal excitability were found to be highest (…) following stronger cortical responses to heartbeats". This sentence is ambiguous as cortical activity is mentioned twice (as the source and the consequence).*

In response to the reviewer's comment, we have revised the sentence to prevent ambiguity.

Cortical and corticospinal excitability were found to be highest during systole and following stronger neural responses to heartbeats. [page 2, line 26-27]

3.3 *In the introduction section, the authors stipulated that "What remains unknown are the underlying mechanisms of these effects". The authors could be more specific to highlight the goal and the originality of their study, to explain the opposite results.*

To address this issue, we revised this section of the introduction:

An overall systolic dampening of cortical processes was suggested to be due to baroreceptor activation during systole (Duschek et al., 2013). In support of this view, reaction times to auditory, visual and tactile stimuli have been shown to be slower for systolic presentation (Edwards et al., 2007; Saari & Pappas, 1976). Furthermore, when touch was initiated during systole, the duration of active sensation was longer (Galvez-Pol et al., 2022). However, for some other motor activities, a facilitatory effect of systole has been observed, e.g., self-initiated movements occurred more frequently during systole (Galvez-Pol et al., 2022; Kunzendorf et al., 2019) or around the R-peak (Palser et al., 2021); but see (Herman & Tsakiris, 2020). Similarly, an effect of the cardiac cycle was also observed in gun shooting, with shooters preferentially triggering a gun in a cardiac window that included a large part of systole and the initial part of diastole (Konttinen et al., 2003); but also see an opposite effect for elite shooters (Helin et al., 1987). Furthermore, (micro)saccades occur more often during systole whereas fixations during diastole (Galvez-Pol et al., 2020; Ohl et al., 2016). One potential explanation for these effects has been suggested to be related to blood movement within the body (peripheral), rather than being driven by top-down neural mechanisms (central) (Galvez-Pol et al., 2020). In addition to cardiac phase effects, stronger neural responses to heartbeats, i.e., heartbeat-evoked potentials, are followed by increases in visual perception and decreases in somatosensory detection (Al et al., 2020, 2021; Park, Correia, Ducorps, & Tallon-Baudry, 2014). These results therefore suggest that cardiac activity could interact with both perception and action. However, it is not known whether a central/cortical mechanism underlies these cardiac effects. [page 2-3, line 41-58]

3.4 *It is not clear if the authors synchronized the TMS pulses and the cardiac phases or if they stimulated, then categorized a posteriori in which cycle the TMS pulse was triggered.*

We thank the reviewer for this question. TMS stimulation was administered at random times throughout the cardiac cycle, and the categorization of TMS pulses into specific cardiac phases was done after the stimulation. We purposely chose this approach to overcome the limitations of classical methods that only target specific time points of the cardiac cycle (such as R+200, R+400 ms (Otsuru et al., 2020). Such approaches may not account for the variability of heart rate within and between individuals, potentially resulting in inconsistent interpretations of the effects (e.g., R+400 might correspond to diastole for shorter cardiac cycles, but for others it might be systole). Additionally, by considering the entire cardiac cycle, we were able to analyze the later parts of diastole beyond the commonly examined time points. Our adaptive definition of systole and diastole on a trial-by-trial basis allowed us to capture the phasic effects of the cardiac cycle more comprehensively.

We edited the result section to clarify this point:

We first aimed to determine whether corticospinal excitability changes systematically over the phases of the cardiac cycle. For this purpose, we applied TMS to the right primary motor area at random time points across the cardiac cycle and recorded motor-evoked potentials (MEPs) of the first dorsal interosseus muscle in the left hand in thirty-six participants. Following the TMS administration, the corresponding cardiac phase was determined posteriori (*see Methods for the details).* [page 6, line 119-124]

3.5 *l.172 "significant increase in the normalized EMG envelope". Normalized to what?
    Did the authors use the force produced as a covariable? As it may have influenced the EMG signal.*

We thank the reviewer for raising an ambiguous part in the Results section. The EMG envelope was normalized by dividing it by the peak muscle activation value during each trial, however we realized that this approach was problematic as this results in each cardiac phase to be normalized independently. Additionally, normalization process is generally used to compare EMG results from different muscle groups or different populations. As we have conducted all of our analysis within subject for the same muscle group, we did not need normalization process in our analysis. Therefore, we removed this step from our analysis and revised the manuscript accordingly. In addition, we did not use force as a covariate but asked the subjects to apply 30% of their maximal force. Subjects could see the applied force on the dynamometer, and before starting the task, they practiced applying similar forces across different trials. Please see the revised version of the manuscript below:

To estimate peripheral muscle force, we calculated the envelope of EMG activity when subjects initiated the pinch during systole and diastole (*see EMG Envelope in the Methods*). Cluster statistics revealed a significant increase in the EMG envelope from 340 to 454 ms after the onset of the pinch during systole compared to diastole (*p*_cluster_ =0.02, Cohen’s d=0.44, **Fig. 4a**). [page 8-9, line 184-189]

EMG Envelope

To estimate muscle activity during the pinch task, EMG data were analyzed. First, EMG signal was filtered by using a 4^th^ order Butterworth bandpass filter (10-500 Hz) and a 50 Hz notch filter (with a stopband of 45-55 Hz). Afterwards, the envelope of EMG was calculated by first taking the absolute value of the signal (“full-wave rectification”) and then applying an 8 Hz low-pass filter(Winter, 2009). The resulting EMG envelope was epoched between -1 and 4 s around the movement onset. This was followed by a baseline correction using the -110 to -10ms pre-movement EMG signal. Finally, the envelope was averaged for trials where movement was initiated during systole and diastole per subject. [page 26, line 681-688]

3.6 *l.180 "we also tested whether sensorimotor oscillations (in the range of 8 - 30 Hz)" "the desynchronization of sensorimotor oscillations in the range of 8 - 25 Hz was stronger". Why this range? And why is the range different (8-30; 8-25)?*

We thank the reviewer for the question. We tested sensorimotor oscillations in the range from 8 to 30 Hz when a movement was initiated during systole as compared to diastole using cluster statistics (which was done in 3 dimensions: time x space x frequencies). This test revealed a significant cluster extending from 8to25 Hz. Since we noticed that this writing style can be ambiguous, we removed the info regarding 8–30 Hz from results but rather give this information in the methods section only:

Results

Following the analysis of the muscle activity in the periphery, we also tested whether sensorimotor oscillations in the motor areas desynchronize differently following the initiation of the pinch during systole and diastole. This analysis demonstrated that the desynchronization of sensorimotor oscillations in the range of 8–25 Hz was stronger between 0 and 726 ms following pinch onset during systole as compared to diastole. [page 9, line 195-199]

Methods

During the motor task, the statistical analysis focused on the first second of the muscle contraction following the pinch onset, as cardiac effect are expected to be transient and may last for one cardiac cycle only. Therefore, statistical analysis of the EMG envelope during systole and diastole were conducted in a time window from 0 to 1000 ms. During the same time window, sensorimotor oscillations were compared in the range from 8 to 30 Hz over a set of electrodes over sensorimotor regions (C4, CP4, C6, CP6) using cluster statistics, in order to account for multiple comparisons in the temporal, spatial and frequency domain. [page 28, line 727-733]

3.7 *l.234: "we sorted single trials according to their MEP amplitudes and split them into three equal bins for each participant". Why did the authors choose 3 bins (and not 2 or 4)?*

We sorted trials into 3 bins since it allowed us to have a strong contrast between weak and strong MEPs while providing a sufficiently high number of trials in each category. To explain our rationale, we edited the ms.

For statistical testing of HEP activity relating to motor excitability, we first sorted single trials according to their MEP amplitudes and split them into three equal bins for each participant. We specifically used three bins to create a strong contrast between weak and strong MEPs while providing a sufficiently high number of trials in each category. [page 27, line 716-719]

3.8 *From l. 203 and l.255: it is not clear why the authors investigated "the changes in the heart rate in response to TMS stimulation" and "the effect of motor excitability on heart rates". After focusing on the modulations of central and peripheral changes across the cardiac cycles, the authors analyzed the other way around. They should clarify why these analyses were important to answer the general problematic.*

We greatly appreciate the reviewer's valuable comment, which has helped us to further improve the manuscript. We edited the introduction and results sections to prepare the reader for upcoming analyses related to the effect of TMS on heart rate, which was missing in the initial version of the manuscript.

Introduction

In addition, in an exploratory analysis, we found a specific influence of TMS on heart rate depending on the cardiac phase, which could be relevant for clinical studies. [page 4, line 96-98]

Results

Previous research suggests that TMS stimulation has significant effects on heart rate (Iseger et al., 2021). However, it is not known whether this effect depends on the cardiac phase of the TMS stimulation. Therefore, in an exploratory analysis, we investigated the changes in the heart rate in response to TMS stimulation during systole and diastole. [page 10, line 219-222]

Additionally, after evaluating overall feedback from the reviewers, we decided that the analysis of heart rate in relation to motor excitability was not well-connected with the rest of the manuscript and seemed to distract the reader from the main questions. Therefore, we removed this section from the manuscript to simplify it.

3.9 *L.260 "as motor excitability increased, the heartbeats became faster (cardiac acceleration)". Could we also say that "as heartbeats became faster, motor excitability increased"? How can the authors interpret the causality between heartbeat and MEP amplitude modulation? And I would say corticospinal or corticomotor (not just motor) excitability when interpreting MEP amplitude modulation.*

The reviewer is right. The sentence is misleading. After evaluating overall feedback from the reviewers, we decided that this analysis distracts the reader from the main questions. Therefore, we removed this section from the manuscript to simplify it.

3.10 *l.366 "Overall, these results suggest that motor excitability is higher during systole, suggesting an optimal window for motor activity across the cardiac cycle". Could the authors be more specific about this argument? Do the small (but significant) differences in cortical and corticospinal excitability across the cardiac cycles prevail for functional efficiency?*

In order to discuss our statement in the context of actual functional benefits, we have included the following part:

Overall, we found that the excitability in the sensorimotor cortex is increased in systole and correspondingly such increase is mirrored in the strength of the motor output. However, the strength of the motor output is not the only parameter important for motor control. The timing of the movement initiation as well as the coordination between different muscle groups could be equally important for the functionally relevant motor output. These aspects should be investigated in future studies. [page 16, line 377-383]

3.11 *l.468: "Given a Cohen's d of 0.48, we calculated the required sample size as N=36 (80% power and alpha=0.05) for observing cardiac effects on perception". Why do the authors refer to 'perception' here?*

We calculated the required sample size for investigating heart-brain interaction using EEG from our previous study (Al et al., 2020) which focused on somatosensory perception. In agreement with the first hypothesis, we expected a similar sample size to work in the motor domain. We took this study as a basis since there were no other EEG studies the examined cardiac cycle effects in the motor domain. As we noticed, this sentence might be confusing for the reader. We revised it as follows:

Based on our previous study in which we observed an effect of the cardiac cycle on somatosensory perception (Motyka et al., 2019), we performed a power analysis (using the "pwr" package in R). Given a Cohen's d of 0.48, we calculated the required sample size as N=36 (80% power and alpha=0.05) for observing cardiac effects of this size. [page 20, line 504-507]

3.12 *l.627: "The trials were categorized into systole and diastole according to the movement onset of the subject after the visual cue". Was the beginning of the contraction synchronized with the cardiac cycles? Or was the categorization performed a posteriori?*

The categorization was done a posteriori. First, we identified the movement onsets by analyzing the EMG data. Then, we determined whether this movement onset occurred during systole or diastole. We thank the reviewer for pointing out this missing information in the methods section. To address this, we have added a new subsection to clarify this procedure:

Movement Onset Estimation

The onset of movement was determined by analyzing EMG activity. For this purpose, we used a Matlab package, EMG-onset-detection. This package uses a method based on Teager-Kaiser Energy (TKE) operator and corrections of false onset detection and EMG artifacts (Yang, Zhang, Gu, & Liu, 2017). If automatic detection did not work, the onset of movement was determined manually. Then, we determined the corresponding cardiac phase of each movement onset. On average, each cardiac phase included twelve trials. [page 26, line 672-678]

3.13 *How many trials during systole? Also, between the presentation of the Go signal, and the beginning of the contraction, there is a reaction time (between 130 and 150ms if the instructions are to move as fast as possible at the imperative signal). How did the authors make sure that the analyzed trial was categorized into the right category? Did they take into account the presentation of the signal or the beginning of EMG activation? EMG activation could begin during systole while the presentation of the signal was during diastole, and inversely.*

We apologize for the missing information in the manuscript. As we indicated in our answer in 3.12, we individually determined movement onsets. On average, twelve trials were included in each cardiac phase. We added this information in the manuscript.

3.14 *l.638: "an average of the envelope was calculated when pinch onset coincided with the systolic and diastolic phases of the cardiac cycle per subject". What was the window for averaging EMG envelope?*

Here, we meant that we averaged EMG envelopes across the movements initiated during systole and diastole. We restricted the analyses of EMG to a time window from 0 to 1 s following movement initiation as the cardiac effects are expected to be transient. We edited the manuscript to ensure that this point is conveyed more clearly:

The resulting EMG envelope was normalized by dividing it by the peak muscle activation value during each trial for the time window from 0 to 1 s following the pinch. This was followed by a baseline correction using the -110 to -10ms pre-movement EMG signal. Finally, the envelope was averaged for trials where movement was initiated during systole and diastole per subject. [page 26, line 685-688]

During the motor task, the statistical analysis focused on the first second of the muscle contraction following the pinch onset, as cardiac effects are expected to be transient and may last for one cardiac cycle only. [page 28, line 727-729] *------------------------------------
EDITORIAL REQUESTS:*

*1) TITLE: After some discussion, we think the title could be strengthened to more directly reflect the findings of your study. If you agree (and if supported), we might suggest it be changed to something like "Systolic cardiac signals facilitate motor excitability'*

We appreciate the suggestion from the editorial office. While we understand the desire to highlight specific findings, we believe that our current title reflects the broader scope of our study, encompassing both heartbeat-evoked potential and heart rate-related findings. While we prefer the title “Cardiac Activity Impacts Cortical Motor Excitability”, we are open to further discussion and would be happy to consider alternative title suggestions.

*2) FINANCIAL DISCLOSURES: I noticed in your financial disclosure that you report having received no specific funding for this work. Even if you did not have a grant specifically written to fund this project, we would require that you acknowledge the sources of money that funded this project, including salaries, etc. You should also acknowledge institutional funding or private grants as well, if relevant.*

For this project, Esra Al received funding from the Max Planck School of Cognition, and the experiment was funded by the Max Planck Society.

*3) ETHICS STATEMENT: Please update your ethics statement to note whether your study was conducted according to the principles expressed in the Declaration of Helsinki.*

We edited the ethics statement to indicate that it followed the principles of the Declaration of Helsinki.

All protocols were approved by the Ethical Committee of the University of Leipzig’s Medical Faculty (Ethics no: 179/19) and followed the principles of Declaration of Helsinki.

*4) BLURB: When re-submitting, please provide a blurb which (if accepted) will be included in our weekly and monthly Electronic Table of Contents, sent out to readers of PLOS Biology, and may be used to promote your article in social media. The blurb should be about 30-40 words long and is subject to editorial changes. It should, without exaggeration, entice people to read your manuscript. It should not be redundant with the title and should not contain acronyms or abbreviations.*

Please see the blurb below.

By studying motor excitability and muscle activity across the cardiac cycle of humans, this study reveals previously unknown heart-brain interactions that suggest distinct time windows optimized for either action or perception.

*5) DATA AVAILABILITY: You may be aware of the PLOS Data Policy, which requires that all data be made available without restriction:* [*http://journals.plos.org/plosbiology/s/data-availability*](http://journals.plos.org/plosbiology/s/data-availability)*. For more information, please also see this editorial:* [*http://dx.doi.org/10.1371/journal.pbio.1001797*](http://dx.doi.org/10.1371/journal.pbio.1001797) 

*Note that we do not require all raw data (we understand that your protocol does not permit the public distribution of the raw data collected in your study). Rather, we ask that all individual quantitative observations that underlie the data summarized in the figures and results of your paper be made available in one of the following forms:*

We have provided the code for creating figures and subject data in the following link: <https://osf.io/4c8s3/?view_only=7edb12b6e50e4c709ad60579bf5ebd62> . Readers can download the code and run the Matlab and R scripts to generate the figures and examine the statistical analyses conducted in the paper. Additionally, the experimental scripts and complete analysis code can be accessed at <https://github.com/Esra-Al/Cardiac_Motor_TMS_EEG> .

*---Please also ensure that figure legends in your manuscript include information on where the underlying data can be found, and ensure your supplemental data file/s has a legend.*

Data and code are provided in the following publicly accessible repository: <https://osf.io/4c8s3/?view_only=7edb12b6e50e4c709ad60579bf5ebd62>. We have included this information in the methods section of the manuscript. Additionally, in the legend of Figure 2, we have included a note stating that all data and code related to the remaining figures can be found at the same link. *---Please ensure that your Data Statement in the submission system accurately describes where your data can be found.*

We added this information in the submission system.

Al, E., Iliopoulos, F., Forschack, N., Nierhaus, T., Grund, M., Motyka, P., … Villringer, A. (2020). Heart-brain interactions shape somatosensory perception and evoked potentials. *Proceedings of the National Academy of Sciences of the United States of America*, *117*(19), 10575–10584. https://doi.org/10.1073/pnas.1915629117

Al, E., Iliopoulos, F., Nikulin, V. V., & Villringer, A. (2021). Heartbeat and somatosensory perception. *NeuroImage*, *238*, 118247. https://doi.org/10.1016/J.NEUROIMAGE.2021.118247

Droste, C., Kardos, A., Brody, S., Greenlee, M. W., Roskamm, H., & Rau, H. (1994). Baroreceptor stimulation: pain perception and sensory thresholds. *Biological Psychology*, *37*(2), 101–113. https://doi.org/10.1016/0301-0511(94)90025-6

Duschek, S., Werner, N. S., & Paso, G. A. R. del. (2013). The behavioral impact of baroreflex function: A review. *Psychophysiology*, *50*(12), 1183–1193. https://doi.org/10.1111/PSYP.12136

Dusi, V., & Ardell, J. L. (2020). Brain-Heart Afferent-Efferent Traffic. *Brain and Heart Dynamics*, 3–24. https://doi.org/10.1007/978-3-030-28008-6_2/COVER

Dworkin, B. R., Elbert, T., Rau, H., Birbaumer, N., Pauli, P., Droste, C., & Brunia, C. H. M. (1994). Central effects of baroreceptor activation in humans: Attenuation ofskeletal reflexes and pain perception. National Academy of Sciences of the United States of America. Retrieved from https://research.tilburguniversity.edu/en/publications/central-effects-of-baroreceptor-activation-in-humans-attenuation-

Edwards, L., Inui, K., Ring, C., Wang, X., & Kakigi, R. (2008). Pain-related evoked potentials are modulated across the cardiac cycle. *PAIN®*, *137*(3), 488–494. https://doi.org/10.1016/J.PAIN.2007.10.010

Edwards, L., Ring, C., McIntyre, D., Carroll, D., & Martin, U. (2007). Psychomotor speed in hypertension: Effects of reaction time components, stimulus modality, and phase of the cardiac cycle. *Psychophysiology*, *44*(3), 459–468. https://doi.org/10.1111/j.1469-8986.2007.00521.x

Fonov, V., Evans, A., McKinstry, R., Almli, C., & Collins, D. (2009). Unbiased nonlinear average age-appropriate brain templates from birth to adulthood. *NeuroImage*, *47*, S102. https://doi.org/10.1016/s1053-8119(09)70884-5

Galvez-Pol, A., McConnell, R., & Kilner, J. M. (2020). Active sampling in visual search is coupled to the cardiac cycle. *Cognition*, *196*. https://doi.org/10.1016/j.cognition.2019.104149

Galvez-Pol, A., Virdee, P., Villacampa, J., & Kilner, J. M. (2022). Active tactile discrimination is coupled with and modulated by the cardiac cycle. *ELife*, *11*. https://doi.org/10.7554/ELIFE.78126

Hannula, H., & Ilmoniemi, R. J. (2017). Basic Principles of Navigated TMS BT - Navigated Transcranial Magnetic Stimulation in Neurosurgery. In S. M. Krieg (Ed.) (pp. 3–29). Cham: Springer International Publishing. https://doi.org/10.1007/978-3-319-54918-7_1

Helin, P., Sihvonen, T., & Hänninen, O. (1987). Timing of the triggering action of shooting in relation to the cardiac cycle. *British Journal of Sports Medicine*, *21*(1), 33–36. https://doi.org/10.1136/BJSM.21.1.33

Herman, A. M., & Tsakiris, M. (2020). Feeling in Control: The Role of Cardiac Timing in the Sense of Agency. *Affective Science 2020 1:3*, *1*(3), 155–171. https://doi.org/10.1007/S42761-020-00013-X

Hernandez-Pavon, J. C., Veniero, D., Bergmann, T. O., Belardinelli, P., Bortoletto, M., Casarotto, S., … Ilmoniemi, R. J. (2023). TMS combined with EEG: Recommendations and open issues for data collection and analysis. *Brain Stimulation*, *16*(2), 567–593. https://doi.org/10.1016/J.BRS.2023.02.009/ATTACHMENT/79C9D8D9-1EFC-4C9A-96A3-EA6B033746FE/MMC1.DOCX

Hyvärinen, A. (1999). Fast and robust fixed-point algorithms for independent component analysis. *IEEE Transactions on Neural Networks*, *10*(3), 626–634. https://doi.org/10.1109/72.761722

Iseger, T. A., Padberg, F., Kenemans, J. L., van Dijk, H., & Arns, M. (2021). Neuro-Cardiac-Guided TMS (NCG TMS): A replication and extension study. *Biological Psychology*, *162*, 108097. https://doi.org/10.1016/J.BIOPSYCHO.2021.108097

Ito, J., Roy, S., Liu, Y., Cao, Y., Fletcher, M., Lu, L., … Heck, D. H. (2014). Whisker barrel cortex delta oscillations and gamma power in the awake mouse are linked to respiration. *Nature Communications 2014 5:1*, *5*(1), 1–10. https://doi.org/10.1038/ncomms4572

Kluger, D. S., Balestrieri, E., Busch, N. A., & Gross, J. (2021). Respiration aligns perception with neural excitability. *ELife*, *10*. https://doi.org/10.7554/ELIFE.70907

Konttinen, N., Mets, T., Lyytinen, H., & Paananen, M. (2003). Timing of triggering in relation to the cardiac cycle in nonelite rifle shooters. *Research Quarterly for Exercise and Sport*, *74*(4), 395–400. https://doi.org/10.1080/02701367.2003.10609110

Kunzendorf, S., Klotzsche, F., Akbal, M., Villringer, A., Ohl, S., & Gaebler, M. (2019). Active information sampling varies across the cardiac cycle. *Psychophysiology*, *56*(5), 1–16. https://doi.org/10.1111/psyp.13322

Lacey, J. I., & Lacey, B. C. (1970). SOME AUTONOMIC-CENTRAL NERVOUS SYSTEM INTERRELATIONSHIPS (pp. 205–227). Retrieved from https://www.sciencedirect.com/science/article/abs/pii/B9780121028503500165?via%3Dihub

Lawrence, M. (2016). ez: Easy Analysis and Visualization of Factorial Experiments. Retrieved from https://cran.r-project.org/package=ez

Li, D., Mabrouk, O. S., Liu, T., Tian, F., Xu, G., Rengifo, S., … Borjigin, J. (2015). Asphyxia-activated corticocardiac signaling accelerates onset of cardiac arrest. *Source*, *112*(16), 2073–2082. https://doi.org/10.2307/26462358

Motyka, P., Grund, M., Forschack, N., Al, E., Villringer, A., & Gaebler, M. (2019). Interactions between cardiac activity and conscious somatosensory perception. *Psychophysiology*, e13424.

Nguyen, T., & Davidson, B. P. (2019). Contrast Enhanced Ultrasound Perfusion Imaging in Skeletal Muscle. *Journal of Cardiovascular Imaging*, *27*(3), 163–177. https://doi.org/10.4250/JCVI.2019.27.E31

Nikouline, V., Ruohonen, J., & Ilmoniemi, R. J. (1999). The role of the coil click in TMS assessed with simultaneous EEG. *Clinical Neurophysiology : Official Journal of the International Federation of Clinical Neurophysiology*, *110*(8), 1325–1328. https://doi.org/10.1016/S1388-2457(99)00070-X

Nyklíček, I., Wijnen, V., & Rau, H. (2005). Effects of baroreceptor stimulation and opioids on the auditory startle reflex. *Psychophysiology*, *42*(2), 213–222. https://doi.org/10.1111/J.1469-8986.2005.00273.X

Ohl, S., Wohltat, C., Kliegl, R., Pollatos, O., & Engbert, R. (2016). Microsaccades are coupled to heartbeat. *Journal of Neuroscience*, *36*(4), 1237–1241. https://doi.org/10.1523/JNEUROSCI.2211-15.2016

Otsuru, N., Miyaguchi, S., Kojima, S., Yamashiro, K., Sato, D., Yokota, H., … Onishi, H. (2020). Timing of Modulation of Corticospinal Excitability by Heartbeat Differs with Interoceptive Accuracy. *Neuroscience*, *433*, 156–162. https://doi.org/10.1016/J.NEUROSCIENCE.2020.03.014

Palser, E. R., Glass, J., Fotopoulou, A., & Kilner, J. M. (2021). Relationship between cardiac cycle and the timing of actions during action execution and observation. *Cognition*, *217*. https://doi.org/10.1016/J.COGNITION.2021.104907

Park, H.-D., & Blanke, O. (2019). Heartbeat-evoked cortical responses: Underlying mechanisms, functional roles, and methodological considerations. *NeuroImage*, *197*, 502–511. https://doi.org/10.1016/j.neuroimage.2019.04.081

Park, H.-D., Correia, S., Ducorps, A., & Tallon-Baudry, C. (2014). Spontaneous fluctuations in neural responses to heartbeats predict visual detection. *Nature Neuroscience*, *17*(4), 612–618. https://doi.org/10.1038/nn.3671

R Development Core Team. (2021). R: A Language and Environment for Statistical Computing. Vienna, Austria.

Rae, C. L., Ahmad, A., Larsson, D. E. O., Silva, M., Praag, C. D. G. van, Garfinkel, S. N., & Critchley, H. D. (2020). Impact of cardiac interoception cues and confidence on voluntary decisions to make or withhold action in an intentional inhibition task. *Scientific Reports*, *10*(1). https://doi.org/10.1038/S41598-020-60405-8

Rau, H., & Elbert, T. (2001). Psychophysiology of arterial baroreceptors and the etiology of hypertension. *Biological Psychology*, *57*(1–3), 179–201. Retrieved from http://www.ncbi.nlm.nih.gov/pubmed/11454439

Rogasch, N. C., Sullivan, C., Thomson, R. H., Rose, N. S., Bailey, N. W., Fitzgerald, P. B., … Hernandez-Pavon, J. C. (2017). Analysing concurrent transcranial magnetic stimulation and electroencephalographic data: A review and introduction to the open-source TESA software. *NeuroImage*, *147*, 934–951. https://doi.org/10.1016/J.NEUROIMAGE.2016.10.031

Rossini, P. M., Barker, A. T., Berardelli, A., Caramia, M. D., Caruso, G., Cracco, R. Q., … Tomberg, C. (1994). Non-invasive electrical and magnetic stimulation of the brain, spinal cord and roots: basic principles and procedures for routine clinical application. Report of an IFCN committee. *Electroencephalography and Clinical Neurophysiology*, *91*(2), 79–92. https://doi.org/10.1016/0013-4694(94)90029-9

Saari, M. J., & Pappas, B. A. (1976). Cardiac Cycle Phase and Movement and Reaction Times. *Perceptual and Motor Skills*, *42*(3), 767–770. https://doi.org/10.2466/pms.1976.42.3.767

Shepherd, J. T. (1985). The heart as a sensory organ. *Journal of the American College of Cardiology*, *5*(6), 83B-87B. https://doi.org/10.1016/S0735-1097(85)80533-7

Winter, D. A. (2009). Biomechanics and Motor Control of Human Movement: Fourth Edition. *Biomechanics and Motor Control of Human Movement: Fourth Edition*, 1–370.

Yang, D., Zhang, H., Gu, Y., & Liu, H. (2017). Accurate EMG onset detection in pathological, weak and noisy myoelectric signals. *Biomedical Signal Processing and Control*, *33*, 306–315. https://doi.org/10.1016/J.BSPC.2016.12.014

Zhong, X., Meyer, C. H., Schlesinger, D. J., Sheehan, J. P., Epstein, F. H., Larner, J. M., … Cai, J. (2009). Tracking brain motion during the cardiac cycle using spiral cine-DENSE MRI. *Medical Physics*, *36*(8), 3413–3419. https://doi.org/10.1118/1.3157109
