# Supplementary material for: Cardiac activity impacts cortical motor excitability
Source: PLoS Biol. 2023 Nov 28;21(11):e3002393. doi: 10.1371/journal.pbio.3002393 (PMC10684011; doi:10.1371/journal.pbio.3002393)
Supplement: Attachment — Submitted filename: Response_plosbiology_rev2_final.docx [file pbio.3002393.s005.docx]

**RESPONSE LETTER**

We thank all the reviewers and editors for taking their time and providing a valuable feedback and constructive comments. We are pleased to hear that the revisions have been largely successful. We acknowledge the additional suggestions and concerns raised by the reviewers and we carefully addressed all of them.

In what follows, the reviewers’ comments and editorial requests are in italics and our responses to each comment are in plain text. Quotes from the revised manuscript are printed in blue font color.

**EDITORIAL REQUESTS**

*1) We see that you have provided code and subject data as a deposition to OSF. Thank you for this. Can you please update the OSF link to include a readme file detailing what is contained there and how it relates to the figures?*

Thank you for this suggestion. We added a readme file on the OSF link:

\# README

This repository includes code and data used in the creation of figures and the statistical evaluation of the main results presented in the manuscript titled "Cardiac Activity Impacts Cortical Motor Excitability."

\## Repository Contents

The repository is structured as follows:

- **figures_code:** This directory contains subfolders corresponding to each main and supplementary figure in the manuscript. For figures with subparts, additional subfolders can be found inside the corresponding figure folder. Inside these subfolders, you can access both the data files and relevant R or Matlab code.

\## How to Use This Repository

1. **figures_code:** To replicate or explore the statistical analyses presented in the manuscript, navigate to the " figures_code" directory and choose the folder for the figure or supplementary figure of interest. Inside each figure folder, you will find both the code and data files necessary to reproduce the results. For figures with subparts, navigate to the respective subfolders (e.g., Figure 2a). Open the code of interest and change the variable *in main_path* as the directory of “figures_code”

\## Manuscript Reference

If you use any code or data from this repository in your own work, kindly cite our manuscript.

\## Questions or Assistance

If you have questions or need further information about the repository's contents, please feel free to reach out to us at [esraal@cbs.mpg.de].

Thank you for your interest in our research.

*2) We understand that you are not able to share the raw EEG and MRI data related to your study due to consent issues. This is OK, under our acceptable data restriction policy (https://journals.plos.org/plosone/s/data-availability#loc-acceptable-data-access-restrictions) - but we ask that you please update your data availability statement to provide contact information for a data access committee, ethics committee, or other institutional body to which data requests may be sent (rather than directing researchers to a corresponding author). This provides researchers with a more durable point of contact to ensure data will be accessible even if an author changes email addresses, institutions, or becomes unavailable to answer requests.*

While our institute does not have a data access committee, the following email [info@cbs.mpg.de](mailto:info@cbs.mpg.de) can be used to write to the institute for data access requests. We added this information in the “Data and code availability” section of the manuscript.

If the corresponding author is not reachable for data request, [info@cbs.mpg.de](mailto:info@cbs.mpg.de) can be contacted.

*3) While we understand that you cannot provide the raw data related to your study, I wonder if for some figures you can provide the anonymized summary statistics that underlie the graphs presented here? For example, can you provide, in an excel or on OSF, the individual quantitative observations that underlie the data summarized in the figures 5, S1, S3?*

As it was requested, we provided the individual quantitative observations that underlie the data summarized in the figures 5, S1, S3 as excel files on the OSF link (<https://osf.io/4c8s3/?view_only=7edb12b6e50e4c709ad60579bf5ebd62>) and also added this information to the “Data and code availability” section of the manuscript.

Reviewer #1 - Comments and Responses

1.1 *Al et al. investigated the relationship between cardiac phases (systole and diastole) and cortical as well as corticospinal excitability through an amalgam of techniques: TMS, EEG, and ECG. Elevated excitability was observed during the systole phase, alongside increased EMG activity during a simplistic motor task. These findings potentially indicate a correlation between the systole phase and motor facilitation. Upon reviewing both the preprint and subsequent versions, it is apparent that the authors have judiciously employed diverse methodologies to address salient questions, particularly in the realm of interoception. Overall, the manuscript has been substantially improved, aligning well with prior reviewer feedback. The revisions have enhanced the paper's scope, clarity, and primary conclusions. Minor observations, which are readily addressable, are as follows:*

We thank the reviewer for the positive and constructive feedback. We addressed the remaining concerns and changed the manuscript accordingly. Please find our responses to each comment below.

1.2 *The results indicating increased excitability are modest, with a p-value approximating 0.040. Consequently, I recommend circumspect language in both the abstract and discussion where these findings are discussed. For example, in the abstract, the phrase "Cortical and corticospinal excitability were found to be highest during systole" could be cautiously reworded. Additionally, consideration should be given to the use of words like "may" in statements like "distinct time windows 'may' exist across the cardiac cycle that either optimize perception or action.". Overall please revise the sentences along the manuscript where absolute/superlative terminology are used to state the results of excitability. Also, noting at least once that such results yielded modest significance would give a transparent and clear image of the present work.*

The reviewer is correct in noting that the p-value of the difference in corticospinal excitability, measured by MEP levels, between systole and diastole is close to 0.04. It is important to clarify that we used a significance level of 0.05 in our analysis, and while the p-value is close to this threshold, it nonetheless indicates a significant difference. This indicates that corticospinal excitability is significantly higher during systole in comparison to diastole. We also provide additional evidence supporting this finding in Supplementary Figure 3. Furthermore, in line with the MEP results, we have observed that also *cortical* excitability is significantly higher during systole in comparison to diastole (p = 0.008), as tested by TEPs in the motor cortex. Both of these results jointly suggest that motor excitability is heightened during systole. Given the short nature of the abstract, we suggest keeping the original sentence ("Cortical and corticospinal excitability were found to be highest during systole"). Yet following the suggestion of the reviewer, in the discussion, we emphasize that while the influence of cardiac phase on corticospinal excitability is rather modest, its impact on cortical excitability is notably stronger:

Since the observed MEP effect was rather small in our study, a larger set of participants (as in the present study) would indeed facilitate a statistical detection of differences. Finally, previous studies only tested cardiac effects on peripheral MEPs without concurrent cortical recordings. Yet, it is known that peripheral MEPs reflect excitability changes at both cortical and spinal-cord levels and special measures should be taken (such as H-reflex) in order to disentangle excitability changes between these two levels (Morita, Baumgarten, Petersen, Christensen, & Nielsen, 1999). On the other hand, TEPs directly reflect changes in cortical excitability, especially at early latencies (Harquel et al., 2016). This can also explain why we observed a stronger effect size for the cardiac modulation of TEPs as compared to MEPs.

In light of the reviewer's second suggestion, we agree with them. Therefore, we have revised the abstract and discussion to eliminate the use of 'may' in the context of these findings:

Thus, it is possible that distinct time windows exist across the cardiac cycle, optimizing either perception or action.

Thus, we propose that optimal windows for action and perception are likely to differ across the cardiac cycle.

1.3 *The manuscript's discussion section, similar to the introduction and results in the earlier version, suffers from a disordered flow of ideas. Adding subheadings to delineate different content types (e.g., Cortical Excitability and Cardiac Cycle, Brain Activity in Systole and Diastole, Further Research and Limitations, etc.) may enhance readability and engagement. Otherwise, the discussion is sometimes read as a bit of chaotic amalgam of ideas/results.*

We thank the reviewer for this suggestion. As they suggested, we included subheadings in the discussion section:

**Importance of methodology for current findings**

**Central and peripheral changes of motor excitability across the cardiac cycle**

**Underlying mechanisms and the link to perception**

**Possible clinical relevance of heart rate changes following TMS stimulation**

**Relationship between heartbeat-evoked potentials and motor excitability levels**

**Limitations**

**Concluding Remarks**

1.4 *The study by Galvez-Pol et al. (2020) mentioned that pulsations due to blood circulation could contribute to observed effects of the cardiac cycle on cognition but did not delve/research this aspect. When referencing this idea in the text, a more nuanced statement or even deleting such statement would be prudent. Otherwise it seems that they (Galvez-Pol) studied such a research question and gave a result accordingly.*

We edited this sentence to highlight that this argument is a possible explanation for their findings but was not directly studied in Galvez-Pol et al.

One possible explanation for these effects can be related to blood movement within the body (peripheral), rather than being driven by top-down neural mechanisms (central).

*Reviewer # 2 - Comments and Responses*

2.1 *The authors have clarified the manuscript making it more accessible and the structure now better supports also the understanding of the different data types. All my questions has been thoroughly answered and when relevant manuscript modified accordingly.*

We thank the reviewer for the positive feedback.

2.2 *As a minor puzzling point: the rationale/motivation for including the TMS induced effects in heart rate is still not clear to me based on the introduction; the added sentence only mentions it is included leaving the topic rather separate from the rest of the introduction. However, in the discussion, heart rate results are elaborated, making it clearer and more understandable how this analysis/section link with the overall scope of the study. I wonder if it would be possible to add some more reasoning from this perspective to introduction. This issue does not require re-review.*

We edited our introduction to address this issue as follows:

Additionally, to comprehensively evaluate cardiac-related activity, we investigated variations in heart rate following TMS stimulation throughout the cardiac cycle.

2.3 *Typo: line 158-159; neuronal responses e by TMS, it should not be present in the sham condition.*

We thank the reviewer for this correction. We edited the sentence:

Therefore, if the cardiac cycle effect on TEPs is a genuine modulation of evoked neuronal responses to TMS, it should not be present in the sham condition.

*Reviewer # 3 - Comments and Responses*

3.1 *Florent Lebon (note, this reviewer has signed their review): The authors responded point-by-point to my remarks. While they made substantial modifications within the manuscript to provide a clear message, I still have few general concerns, which could be easily addressed.*

We thank the reviewer for the positive feedback.

3.2 *I do not know the policy of the journal, but it seems odd to provide the results at the end of the introduction section (l.82-89), without any further information about the protocol. I leave the editors decide whether the authors should keep the structure as it is.*

We appreciate the reviewer's feedback regarding the placement of results at the end of the introduction section. We followed a format similar to some other papers published in Plos Biology (such as Mahmoodi, Nili, Bang, Mehring, & Bahrami, 2022; Martínez-Cañada, Ness, Einevoll, Fellin, & Panzeri, 2021). However, we understand that journal policies may vary. We are open to adjusting the manuscript structure following the editors' recommendations. If it is preferred to remove the summary of results from the introduction section, we will gladly make the necessary changes. Otherwise, we would prefer to keep the current structure of the *Introduction*. This should facilitate the understanding of the upcoming *Methods* and *Results* sections.

3.3 *In the current form, the result section looks like an accumulation of analyses with a lack of coherence between the different sub-sections. While the two first paragraphs ("Motor-evoked potentials change across the cardiac cycle", and "TMS-evoked potentials vary across the cardiac cycle") are obvious to respond to the main hypothesis, the other parts (e.g., "Muscle-related peripheral and central activity fluctuates across the cardiac cycle", "Heart rate changes depending on the timing of TMS across the cardiac cycle") are less likely to provide a clear message. I suggest the authors to provide a short sentence at the beginning of each result sub-section, explaining why this is important to perform these analyses. This will help the reader understand the common thread.*

Thank you for this suggestion. Indeed, we already provided the reasoning for these sections in the results section:

**Muscle-related peripheral and central activity fluctuates across the cardiac cycle**

To determine whether changes in motor excitability during systole facilitate muscle activity in the absence of TMS stimulation, we analyzed cardiac effects on muscle activity in a follow-up motor pinch task. During the task, participants were asked to pinch a dynamometer with their index finger and thumb, while we recorded EMG, EEG, and ECG signals.

**Heart rate changes depending on the timing of TMS across the cardiac cycle**

Previous research suggests that TMS stimulation has significant effects on heart rate (Iseger, Padberg, Kenemans, van Dijk, & Arns, 2021). However, it is not known whether this effect depends on the cardiac phase of the TMS stimulation. Therefore, in an exploratory analysis, we investigated the changes of the heart rate in response to TMS stimulation during systole and diastole

3.4 *Is there any relationship between the paragraphs "Heart rate changes depending on the timing of TMS across the cardiac cycle" line 203 and "Heart rate fluctuates depending on motor excitability levels" line 255? Both deal with "changes in heart rate due to the cardiac timing of TMS". Is there any chance that both paragraphs could be merged? Or at least one after the other?*

In the modified version of the manuscript, we did not include “Heart rate fluctuates depending on motor excitability levels". Thus, there is no more confusion relating to these sentences.

3.5 *The paragraph lines 415-430 should be revised. In rehabilitation, repetitive TMS is used to improve the condition of the patients. Single-pulse TMS (as the method used in this study) provides information about a neurophysiological status at the time of the stimulation. Repetitive TMS includes blocks of stimulation, for which the duration is between 3 and 15 minutes. In the article by Johansen-Berg et al. (reference 45), single-pulse TMS is used to estimate the involvement of specific brain in regions in motor control comparing healthy individuals and stroke patients. I would suggest the authors to revise this perspective section, focusing on the evaluation (single or paired-pulse TMS) and not (or less) the treatment (rTMS).*

We revised this paragraph to highlight that our results were obtained using single pulse TMS to prevent any confusion:

Cortical excitability changes have been associated previously with epilepsy, disorders of consciousness, stroke, and depression (Blumberger et al., 2018; Johansen-Berg et al., 2002). To counterbalance these abnormalities in cortical excitability, therapeutic applications of TMS have been introduced, e.g., for treating depression (Blumberger et al., 2018) or facilitating motor recovery during neurorehabilitation in stroke (Johansen-Berg et al., 2002). Our results on cardiac modulations of cortical excitability raise some important questions for these clinical populations using different TMS protocols. For example, it remains unknown how cortical excitability over the cardiac cycle is modulated in those pathological conditions. Furthermore, our observation that TMS induces changes of the heart rate during systole, i.e., when the cortical processing of heartbeats occurs, but not during diastole, can have important implications for the clinical use of TMS. For instance, in case the changes in heart rate during TMS application are of clinical concern for patients, our results, based on single pulse TMS, suggest that stimulation during diastole may help mitigate these undesired effects. Addressing these questions in future research could provide valuable insights for patients undergoing TMS in clinical settings.

3.6 *P.2, l.48 : delete « . , » before « though »*

In the current version of the manuscript, we corrected this typo.

**References**

Blumberger, D. M., Vila-Rodriguez, F., Thorpe, K. E., Feffer, K., Noda, Y., Giacobbe, P., … Downar, J. (2018). Effectiveness of theta burst versus high-frequency repetitive transcranial magnetic stimulation in patients with depression (THREE-D): a randomised non-inferiority trial. *Lancet*, *391*(10131), 1683–1692. https://doi.org/10.1016/S0140-6736(18)30295-2

Harquel, S., Bacle, T., Beynel, L., Marendaz, C., Chauvin, A., & David, O. (2016). Mapping dynamical properties of cortical microcircuits using robotized TMS and EEG: Towards functional cytoarchitectonics. *NeuroImage*, *135*, 115–124. https://doi.org/10.1016/J.NEUROIMAGE.2016.05.009

Iseger, T. A., Padberg, F., Kenemans, J. L., van Dijk, H., & Arns, M. (2021). Neuro-Cardiac-Guided TMS (NCG TMS): A replication and extension study. *Biological Psychology*, *162*, 108097. https://doi.org/10.1016/J.BIOPSYCHO.2021.108097

Johansen-Berg, H., Rushworth, M. F., Bogdanovic, M. D., Kischka, U., Wimalaratna, S., & Matthews, P. M. (2002). The role of ipsilateral premotor cortex in hand movement after stroke. *Proceedings of the National Academy of Sciences of the United States of America*, *99*(22), 14518–14523. https://doi.org/10.1073/PNAS.222536799

Mahmoodi, A., Nili, H., Bang, D., Mehring, C., & Bahrami, B. (2022). Distinct neurocomputational mechanisms support informational and socially normative conformity. *PLOS Biology*, *20*(3), e3001565. https://doi.org/10.1371/JOURNAL.PBIO.3001565

Martínez-Cañada, P., Ness, T. V., Einevoll, G. T., Fellin, T., & Panzeri, S. (2021). Computation of the electroencephalogram (EEG) from network models of point neurons. *PLOS Computational Biology*, *17*(4), e1008893. https://doi.org/10.1371/JOURNAL.PCBI.1008893

Morita, H., Baumgarten, J., Petersen, N., Christensen, L. O. D., & Nielsen, J. (1999). Recruitment of extensor-carpi-radialis motor units by transcranial magnetic stimulation and radial-nerve stimulation in human subjects. *Experimental Brain Research*, *128*(4), 557–562. https://doi.org/10.1007/S002210050881
